# Phenome-wide association network demonstrates close connection with individual disease trajectories from the HUNT study

**DOI:** 10.1371/journal.pone.0311485

**Published:** 2024-12-27

**Authors:** Martina Hall, Marit K. Skinderhaug, Eivind Almaas

**Affiliations:** 1 Department of Biotechnology and Food Science, NTNU - Norwegian University of Science and Technology, Trondheim, Norway; 2 K. G. Jebsen Center for Genetic Epidemiology, NTNU - Norwegian University of Science and Technology, Trondheim, Norway; International Medical University, MALAYSIA

## Abstract

Disease networks offer a potential road map of connections between diseases. Several studies have created disease networks where diseases are connected either based on shared genes or Single Nucleotide Polymorphism (SNP) associations. However, it is still unclear to which degree SNP-based networks map to empirical, co-observed diseases within a different, general, adult study population spanning over a long time period. We created a SNP-based phenome-wide association network (PheNet) from a large population using the UK biobank phenome-wide association studies. Importantly, the SNP-associations are unbiased towards much studied diseases, adjusted for linkage disequilibrium, case/control imbalances, as well as relatedness. We map the PheNet to significantly co-occurring diseases in the Norwegian HUNT study population, and further, identify consecutively occurring diseases with significant ordering in occurrence, independent of age and gender in the PheNet. Our analysis reveals an overlap far larger than expected by chance between the two disease networks, with diseases typically connecting within their own category. Upon examining the sequential occurrence of diseases in the HUNT dataset, we find a giant component consisting of mostly cardiovascular disorders. This allows us to identify sequentially occurring diseases that are genetically linked and co-occur frequently, while also highlighting non-sequential diseases. Furthermore, we observe that survivors of severe cardiovascular diseases subsequently often face less severe conditions, but with a reduced time until their next fatal illness. The HUNT sub-PheNet showing both genetically and co-observed diseases offers an interesting framework to study groups of diseases and examine if they, in fact, are comorbidities. We find that the HUNT sub-PheNet offers the possibility to pinpoint exactly which mutation(s) constitute shared cause of the diseases. This could be of great benefit to both researchers and clinicians studying relationships between diseases.

## Introduction

Since the inception of the first successful Genome-Wide Associations Study (GWAS) in 2007 [1], a wealth of scientific research over the past 15 years has aimed to identify candidate genetic loci that modulate the risk of developing specific diseases or phenotypes. This cumulative effort has resulted in the GWAS catalog, which now features more than 5,800 publications and about 398,000 associations between Single Nucleotide Polymorphisms (SNPs) and various diseases [2, 3].

In the pursuit of more comprehensive post-GWAS analyses, phenome-wide association studies (PheWAS) have come to the fore. These studies have efficiently mapped out the pleiotropic effects of disease SNPs across a broad array of physiological and clinical outcomes, leveraging the information housed in Electronic Health Records (EHR) [4–6]. The potential of PheWAS analyses extends beyond data interpretation; they present a promising opportunity for a system-level exploration of disease interactions, enhancing our understanding of disease complexity and interconnectedness.

Network medicine exemplifies such a system-level approach. This field was pioneered by Goh et al., who developed the Human Disease Network (HDN) based on causal genes drawn from the Online Mendelian Inheritance in Man (OMIM) database [7]. The HDN interconnects diseases that share one or more associated genes, providing a comprehensive map of known human genetic disorders. Notably, their work revealed that the majority of diseases are interlinked through shared genetic origins [7].

Building on the successful methodology of the HDN, subsequent research has aimed to construct comparable disease networks. Instead of using causal genes, these studies leveraged detailed SNP-disease connections from GWAS to explore disease-disease associations at a genomic level [8–11]. While these studies succeeded in grouping similar diseases based on common genetic SNP findings from GWAS, they encountered limitations. These included a small number of SNPs, participants, or phenotypes; association tests that did not account for imbalanced case-control ratios or relatedness; and potential biases introduced when merging summary statistics from multiple studies. Sriram and co-workers overcome some of these limitations in their case study using UKBB PheWAS when creating the NETMAGE [12] approach, and the authors comment that it would be of benefit to explore the disease links with co-occurrence in a *different* study population.

Several studies have explored the overlap between SNP-based disease networks and observed comorbidity [13, 14]. Both of these investigations leverage robust EHR-data from UKBB participants covering a diverse range of diseases with large samples sizes. While Dong et al utilized a full disease network, their approach was limited by the use of linear mixed model association testing, which is sub-optimal for binary data. In contrast, Sriram and coworkers used summary statistics based on association testing with SAIGE (Scalable and Accurate Implementation of GEneralized mixed model), which is better suited for handling imbalanced case-control ratios and relatedness for binary data [15]. However, their focus was on ego-centric networks (networks focused on a specific node and only showing links from this node to the other nodes, i.e. not showing interactions between the other nodes) for obstetric disorders rather than a full disease network. The analyses of both studies on the overlap between the disease networks and observed comorbidity are, however, limited when the observed comorbidity is based on the same participants as used to create the disease network. To validate the hypotheses that genetically linked diseases are indeed at an increased risk of co-occurrence, it would be necessary to corroborate these links within a different study population.

Unlike previous studies, we construct and study a disease network based on a single-source unbiased PheWas-dataset with the main aim of investigating how many, if any, parts of the genetically linked disease network are actually co-observed within a different study population, that of the Trøndelag Health Study (HUNT). The HUNT study, one of the most extensive longitudinal population studies to date, captures approximately 90% of the adult population in Nord-Trøndelag, Norway from 1984 to 2019 [16, 17]. By matching these participants with Electronic Health Records (EHR) and the Cause of Death registry, we have an almost complete health record history for 90, 103 participants diagnosed with one or more conditions from the PheWAS dataset, spanning August 1987 to June 2017. To our understanding, this represents the most expansive time interval for medical history ever collected from both hospital and general practitioners.

We employ the same UK Biobank-based PheWAS summary statistics as Sriram et al. used to construct ego-centric networks [14], but we expand upon this approach to create a comprehensive disease network with adjusted criteria for phenotype inclusion and SNP associations (see Methods for details). By filtering this network for diseases that do not exhibit strong co-occurrence within the HUNT study population, we highlight the intricate interplay between these two networks. We further show that a substantial portion of the genetically linked diseases are indeed true comorbidities, and we study their temporal ordering. These networks hold significant biological and clinical implications, as they offer valuable insights into disease interconnections. Furthermore, they pave the way for improved disease prevention strategies and potentially inform more effective treatments.

## Materials and methods

### Datasets

#### UK Biobank PheWAS

We create the phenome-wide association network (PheNet) from summary statistics of 1, 403 binary phenotypes from a broad EHR-based PheWAS of ∼400, 000 White British participants of European ancestry [15, 18]. The phenotypes are represented as phenotype codes (phenocodes) which is a collection of similar ICD billing codes from the EHR, and are classified into 17 disease categories in the phenocode system. The phenocodes follow a hierarchical system, where the higher phenocode levels are broader definitions of the lower phenocodes levels. All patients diagnosed with a level 2 phenocode (lowest level used in this dataset) are by definition also diagnosed with the higher levels 1 and 0 (highest level). The summary statistics were generated using SAIGE, which, unlike many other GWAS association tests, handles unbalanced case/control ratios and relatedness with a generalized linear mixed model, controlling for gender, birth year and four principal components (PC1-PC4) [15]. To include as many links as possible and at the same time assuring that the links are based on strong associations, we use a *p*-value threshold of 10^−6^ and a minor allele frequency (MAF) cutoff of 0.1%. Including only the top hits (SNP with lowest *p*-value within 500 kb for the same phenotype), the UKBB summary statistics consists of 9, 055 SNP associations with 1, 374 phenocodes at a 2-digits level.

#### The HUNT study and related health records

The HUNT study is a population based longitudinal study inviting all adult (age ≥20) inhabitants of Nord-Trøndelag county in Norway to health related questionnaires and clinical measurements in four 11-year time intervals, ranging from 1984 until 2019 [16, 17]. The first study in 1984 (HUNT1) had a participation rate at 89.4% of the inhabitants of Nord-Trøndelag. The next rounds of invitations (HUNT2, HUNT3 and HUNT4) expanded the study to include also short interviews, clinical examinations and biological sampling, as well as expanding the sample population to include those aged 13 − 19. For the last survey (HUNT4), also inhabitants of the neighboring Sør-Trøndelag county were included. The uniqueness of the HUNT study is the high participation rate with the ability to follow a large fraction of the population over a time interval of up to 35 years. As of 2020, the HUNT study consists of a total of 230, 000 participants [19].

Another strength of the HUNT study is the possibility to link the participants to several local, regional, and national health related registries due to the unique Norwegian 11-digit personal identification number [16]. Such registries include, among others, the Medical Birth Register of Norway, the Norwegian Prescription Database, the Cancer Register of Norway, the Norwegian Cause of Death Register, and regional (the Nord-Trøndelag Hospital Trust (HNT)) and national (Norway Control and Payment of Health Reimbursement (KUHR)) registers for hospital and general practitioner records.

In this paper, we use data from HUNT1, HUNT2, and HUNT3, and we link the participants to ICD-billing codes from HNT and KUHR and the Norwegian Cause of Death Registry. With this, we have a complete list of diagnoses made at hospital visits (HNT 1987–2017) and at the general practitioner (KUHR 2006–2017) for a total of 90, 103 patients, covering ages at first visit between zero and 100 years (see age and birth year distribution in S1 Fig in S1 File). We are also able to track participants that have died due to the diseases. Since we are interested in the overlap of findings from the UKBB based PheNet, we consider only ICD codes from HUNT that are mapped to phenocodes existing in the PheNet, resulting in 1, 037 of the 1, 374 UKBB phenocodes. Note also here that all patients diagnosed with a level 2 phencode is by definition also diagnosed with the higher levels. The mapping from ICD9 and ICD10 codes were performed using the PheCode Maps [20, 21].

### Construction of the PheNet

The PheWAS summary statistics data shows a list of significant SNP-disease associations, where each SNP is the top SNP with the smallest *p*-value (< 10^−6^) for the disease in a region of ±500 kb. Hence, two diseases can have significant associations in the same region with different SNPs, since one of them may have the smallest *p*-value for one of the diseases and the other may have the lowest *p*-value for the other disease. In order to capture these situations, where different SNPs represent the same associated genetic region, we identify groups of SNPs from the PheWAS summary statistics that are in high LD (*r*^2^ ≥ 0.8) using the *LDmatrix* function from the *LDlinkR* R-library [22, 23] with the European population from the Phase 3 1000 Genome Project. In order to reflect correlated SNPs within the same region, we restrict the analysis to a distance of 500 kb between the SNPs. After applying this step, the PheWAS summary statistics contains a list of associations between diseases and SNPs or SNPs in LD-blocks. In this way, the associations to the diseases are either the unique SNP or loci containing an associated SNP. The PheNet is subsequently generated from this list, where two diseases sharing one or more SNPs or LD-blocks are directly linked in the network. Isolated diseases without any connections to other diseases are excluded from the network and further analysis.

To obtain a reasonable measure of the link weight between pairs of diseases, we utilize the effect sizes of the SNP-disease association, *β*. The effect sizes measures the log odds ratio for obtaining the disease given the presence of the SNP, and is thereby a reasonable measure for the strength of the association between the SNP and the disease. In contrast to using the number of associated SNP/regions or using some merged *p*-value, the effect sizes *β* give information about the genetic effect the SNPs have on the diseases. The *β*- and the *p*-values are results from a regression model testing the association between the given SNP and the disease. While a resulting *p*-value gives evidence for a significant or non-significant association based on the user-specific threshold, the corresponding *β*-value shows the effect of the association.

**Fig 1:**
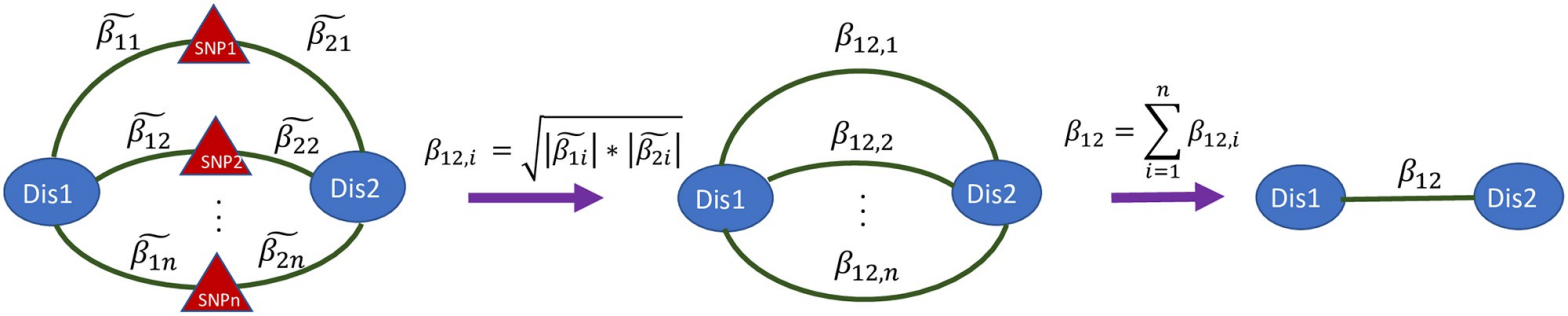
Calculation of link weight for phenotypes sharing common SNPs. The first step involves calculating the geometric mean of the two effect sizes for SNP *i*, ${\tilde{\beta }}_{1,i}$ and ${\tilde{\beta }}_{2,i}$ from the UKBB PheWAS. In the second step, the *n* common SNPs-effects are summed, resulting in the final link weight, *β*_1,2_.

In order to obtain a single link weight between two diseases sharing one or more associated SNPs or LD-blocks we calculate the link weight according to a “mean”-based polygenic risk score (PRS) approach as illustrated in Fig 1. The first step involves capturing the mean effect size linking diseases 1 and 2 though the same SNP or LD-block *i* by merging the two effect sizes, ${\tilde{\beta }}_{1i}$ and ${\tilde{\beta }}_{2i}$, into one by the geometric mean of the absolute effect sizes, $\beta_{12,i}=\sqrt{|{\tilde{\beta }}_{1i}\left|\cdot \right|{\tilde{\beta }}_{2i}|}$. If SNP *i* is a LD-block that is associated with both diseases, we first define ${\tilde{\beta }}_{1i}$ and ${\tilde{\beta }}_{2i}$ as the sum of the SNPs’ absolute effect sizes that are associated to each disease respectively in the LD-block before applying the first steps. Next, with *n* common SNPs/LD-blocks shared between the two diseases, the first step results in *n* mean effect sizes that are then summed into a PRS in the second step. By this, the strength of association, $\beta_{12}=\sum_{i=1}^{n}\beta_{12,i}$, between the two diseases are based on the summed mean effect each SNP has on the two diseases, where diseases with several common SNPs will have a stronger association than diseases with fewer common SNPs, given equivalent effect sizes.

### Overlap with co-occurring diseases in the HUNT study

For each HUNT participant, a list of their registered diseases (considering only disease phenocodes present in the PheNet), are ordered based on each phenocodes first diagnose date. If a person is registered with diseases A, B, C, and D, we construct the pairs A-B, A-C, A-D, B-C, B-D and C-D, where the orderings within each pair is irrelevant. Constructing such pairs for all patients, we count the number of times each pair of diseases are present among the 90, 103 participants. To obtain a measure for the strength of co-occurrence for these disease pairs, we use the Matthews correlation coefficient (MCC) proposed as a comorbidity measure by Hidalgo et. al. [24]. The MCC is a Pearson correlation for binary variables, defined as

$$\begin{array}{c} MCC_{ij}=\frac{C_{ij}N-P_{i}P_{j}}{\sqrt{P_{i}P_{j}\left(N-P_{i}\right)\left(N-P_{j}\right)}}, \end{array}$$ (1)

where *N* is the total number of participants, *C*_*ij*_ is the number of patients with disease *i* and *j*, and *P*_*i*_ and *P*_*j*_ is the number of patients with disease *i* and *j* respectively. To assess only the disease pairs with a co-occurrence larger than expected by chance, we perform a one sided Fisher’s exact test on the contingency table. Extracting only the disease pairs observed in the PheNet, we classify the disease pairs as significantly strong observed comorbidities if the Bonferroni adjusted *p*-value from the one sided Fisher’s exact test is below 0.05/1, 337 = 3.74 ⋅ 10^−5^, where the number 1, 337 is the number of disease pairs tested.

To access the significance of the number of disease pairs from the PheNet showing strong comorbidities in the HUNT population, we compare our finding with the corresponding result from random networks that hold the same network properties as the PheNet. Shuffling the labels (disease names) of the nodes, we simulate 10^4^ random networks, and the number of disease pairs with Bonferroni adjusted significant MCCs are counted for each network. This generates an empirical distribution for the number of significant co-observed pairs in the random networks.

Further, comparing the PheNet disease pairs with the ordered co-occurring disease in the HUNT study population, we perform the same method as above where the disease pairs from the HUNT participants are ordered based on the diagnose date of the disease. A participant with diseases A, B, C and D (in that order) will now give the disease pairs A-B, B-C and C-D. From the constructed frequency list of ordered disease pairs for all participants, the MCCs and corresponding *p*-values are calculated. As the PheNet gives no direction of the links, both directions of the disease pairs are considered when extracting these disease pairs from the frequency list of ordered disease pairs and counting the number of pairs with Bonferroni significant MCCs. This score is again validated against the empirical distribution of corresponding scores from the 10^4^ random networks.

### Creating the HUNT sub-PheNet

The HUNT sub-PheNet is a sub network of the UKBB based PheNet, containing links identified in the PheNet showing strong co-occurrence, defined as Bonferroni adjusted significant MCCs, in the HUNT population. In this way, the HUNT sub-PheNet represents disease associations that are both genetically linked and at the same time being strongly linked as comorbidities based on actual observed disease co-occurrences.

### Network analysis

#### Grouping genetically linked diseases into network modules

As we expect that groups of similar and related diseases will cluster together in the PheNet, we use the Louvain algorithm to construct network modules [25]. This greedy method runs an iterative process of modularity optimization and module aggregation. The modularity measures the density of links within modules compared to links between the modules and is defined as

$$\begin{array}{c} Q=\frac{1}{2m}\;\sum_{ij}[A_{ij}-\frac{k_{i}k_{j}}{2m}]\delta \left(c_{i},c_{j}\right), \end{array}$$ (2)

where *A*_*ij*_ represents the link weight between disease *i* and *j*, *k*_*i*_ = ∑_*j*_ *A*_*ij*_ and *k*_*j*_ = ∑_*i*_ *A*_*ij*_, *m* = ∑_*ij*_*A*_*ij*_, *c*_*i*_ and *c*_*j*_ are the modules of disease *i* and *j*, respectively and *δ* is the Kronecker delta function which is equal to 1 if *c*_*i*_ = *c*_*j*_ and 0 otherwise.

Using the *cluster_louvain* function from the *igraph* R-library [26] we construct modules for the PheNet using the *β*-scores as link weights. The same function is also applied to create modules for the HUNT sub-PheNet with the MCCs are link weights, to investigate the overlap between disease modules based on genetic similarity and disease modules based on strong co-occurrences. Note that the Louvain algorithm does not support negative link weights, but this gives no problems as all *β*-scores are absolute values and all significant MCCs are positive in the HUNT sub-PheNet.

#### Disease homogeneity

In a random network, one would expect that the nearest neighbours of a disease-node (other diseases directly connected to the disease) represent many different categories. In our disease network, we hypothesise that the genetically defined neighbourhood will often belong to the same disease category. In order to quantify the diversity of the disease categories linked to a disease, we calculate a node-based homogeneity score, the H^*^-score [27], for each disease in the network. The H^*^-score is defined as,

$$\begin{array}{c} H_{i}^{*}=\sum_{j=1}^{c}{\left(\frac{k_{ij}}{k_{i}}\right)}^{2} \end{array}$$ (3)

where *k*_*ij*_ is the number of diseases of category *j* linked to disease *i*, *c* is the number of categories, and *k*_*i*_ is the degree of disease *i*. It is hence a function of the number of neighbouring diseases representing the same disease category. If all neighbours of a disease belong to the same disease category, the H^*^-score of the disease will attain its maximum value of unity. If all neighbours have different disease categories, the *H*-score will be at its minimum value close to zero. It is clear from Eq (3) that the H^*^-score is driven by the degree of the disease (number of neighbouring diseases) and hence, also is its minimal value. In order to compare H^*^-values across diseases with different degrees, we scale the H^*^-score according to Ref. [28] to a value between zero and one such that it is independent of the degree of the node. In the following, we will use this scaled H^*^-score. With this, a *H*-score of unity represents a disease whose neighbourhood consists only of the same disease category (including also diseases with only one connection, as the the connected disease category is naturally homogeneous), while a *H*-score of zero represents a neighborhood of maximal difference in disease categories.

To test if the distribution of *H*-scores obtained from the genetically linked diseases in the PheNet is different from what is expected by chance, we compare the mean *H*-scores for all diseases, within each module, and within each disease category, to corresponding mean *H*-scores in 10^4^ random networks holding the same network properties: In each of the simulated networks, only the categories are shuffled without replacement. The distribution of mean *H*-scores for all diseases (and within each module and disease category) is used as an empirical distribution to test the observed *H*-scores from the PheNet. The reported *p*-values are the fraction of random mean *H*-scores larger than the observed *H*-scores after Bonferroni adjustment in the module and category analysis by multiplying with the number of tests (number of modules and number of categories). The same procedure is also applied to the HUNT sub-PheNet, where we investigate if these results from the PheNet still holds when considering a sub-network of diseases that show both genetic and strong co-occurring disease links.

#### Testing interactions across categories with the *Z*-score

To test if some categories are more or less linked to each other than expected by chance, we calculate a *Z*-score, a normalized score for the number of links shared within and between disease categories. Unlike the *H*-score which is a node-based score, the *Z*-score is a link-based measure quantifying the number of interactions within and across categories. It is defined as,

$$\begin{array}{c} x_{i,j}=\sum^{\text{all}\;\text{links}}I\left(\text{link}\;\text{connecting}\;\text{nodes}\;\text{belonging}\;\text{to}\;\text{categories}\;i\;\text{and}\;j\right) \end{array}$$ (4)

$$\begin{array}{c} z_{i,j}=\frac{x_{i,j}-\mu_{i,j}}{\sigma_{i,j}}, \end{array}$$ (5)

where *I*() is an indicator function, *μ*_*i*,*j*_ and *σ*_*i*,*j*_ are the mean and standard deviations of *x*_*i*,*j*_ from 10^4^ random simulated networks, constructed in the same manner as for the *H*-score analysis. The null hypothesis is that the observed number of shared links between each pair of disease categories, *x*_*i*,*j*_, is equal to the corresponding mean of the random networks, *μ*_*i*,*j*_, and the *Z*-score approximates a standard normal distribution. Applying a two sided *Z*-test with a standard normal distribution, we consider *Z*-scores with Bonferroni-adjusted *p*-values < 0.025/136 as significant. Hence, a significant positive/negative *Z*-score for disease categories *i* and *j* indicates that their number of shared links is higher/lower in the genetically-based disease network than expected if the categories were randomly distributed. The *Z*-score analysis is performed for both the PheNet and the HUNT sub-PheNet to investigate if the number of interactions within and between categories change when filtering the network to only include strong co-occurring diseases.

### Direction of disease links and linkage with the Norwegian Cause of Death registry

For the disease links present in the HUNT sub-PheNet that are both genetically linked and often observed together, we investigate their major sequence of occurrence with a two-step approach. First, we question whether or not having disease A gives a higher chance of obtaining disease B when adjusting for age and gender using a regression model. To avoid issues regarding temporal ordering of links within the hierarchical levels of the same phenocode (such as phenocde 174 linked with phenocode 174.1), we consider only disease pairs that are not of higher or lower level of each other in the hierarchical system of the same phenocode. For all such disease pairs in the HUNT sub-PheNet, we apply the following logistic regression model for the association A→ B, testing if the probability of obtaining disease B is higher given that disease A is already present,

$$\begin{array}{c} \text{log}(\frac{P(Y_{ij}=B)}{1-P(Y_{ij}=B)})=\beta_{0}+\beta_{1}\sum_{k=1}^{j-1}I\left(Y_{ik}=A\right)+\beta_{2}Age_{ij}+\beta_{3}Sex_{i}. \end{array}$$ (6)

Here, *Y*_*ij*_ represents the *j*’th disease of individual *i*, *I*(*Y*_*ik*_ = *A*) is a indicator function testing if the *k*’th disease of individual *i* is A, such that summing these up to event *j* will tell if the individual has obtained disease A prior to the *j*’th disease. *Sex*_*i*_ is the gender of the individual and *Age*_*ij*_ is the age of the individual at the *j*’th disease. This model for the A→B association is then fitted for all events up to a possible disease B for all individuals. A positive and significant *β*_1_ will here give evidence for a higher probability of obtaining disease B when disease A is already present, independent of age and gender.

Two such models are fitted for each pair of diseases obtained from the HUNT sub-PheNet, one for each direction. By adjusting for age and gender, we rule out trivial disease progressions, where disease B is subsequent disease A either because of different age of onset or gender, and thus, we identify pairs of subsequent diseases that are independent of age and gender.

From this first step, we extract all directed disease pairs where the *β*_1_ coefficient is positive and significant after Bonferroni adjustment (*p*-value < 0.05/850). Here, both directions of the disease pair are allowed when both models show significant and positive effects for the *β*_1_ coefficient. In the second step, we perform a binomial test to extract the strong and major temporal ordering of disease trajectory pairs identified in step one. In the binomial test, we evaluate the frequency at which disease A precedes disease B (*x*) among individuals diagnosed with both conditions (*n*). The null hypothesis posits that both diseases are equally likely to be observed first, with a probability *p*_*H*0_ = 0.5. Disease trajectory pairs yielding significant *p*-values based on the binomial distribution (Bin(*x*;*n*, *p*)) are deemed to have strong temporal ordering after adjustment for multiple testing using the Bonferroni correction. This suggests a major flow in the disease progression that is independent of age and gender.

Consequently, only the major directional flow of the disease pair is retained after this step. Associations found significant in the regression analysis but with inadequate frequencies in the temporal ordering are eliminated. The median time interval between the occurrences of diseases is recorded for all individuals exhibiting this temporal disease progression. To mitigate potential biases and uncertainties due to limited follow-up durations, only participants with a follow-up period exceeding two years were included in this analysis.

Finally, we link these diseases to the Norwegian Cause of Death registry and determine the mortality rate (number of deaths divided by prevalence of disease) from each of these diseases in the same time span as the hospital and general practitioner records (1987–2017). Note that, in the Norwegian Cause of Death registry, diagnoses highly related to the cause of death are also listed. Also, some participants could have their first event of a specific disease as the cause of death and are hence not registered in the hospital nor general practitioner records with this disease.

## Results

### The PheNet shows genetic linkage between diseases

**Fig 2:**
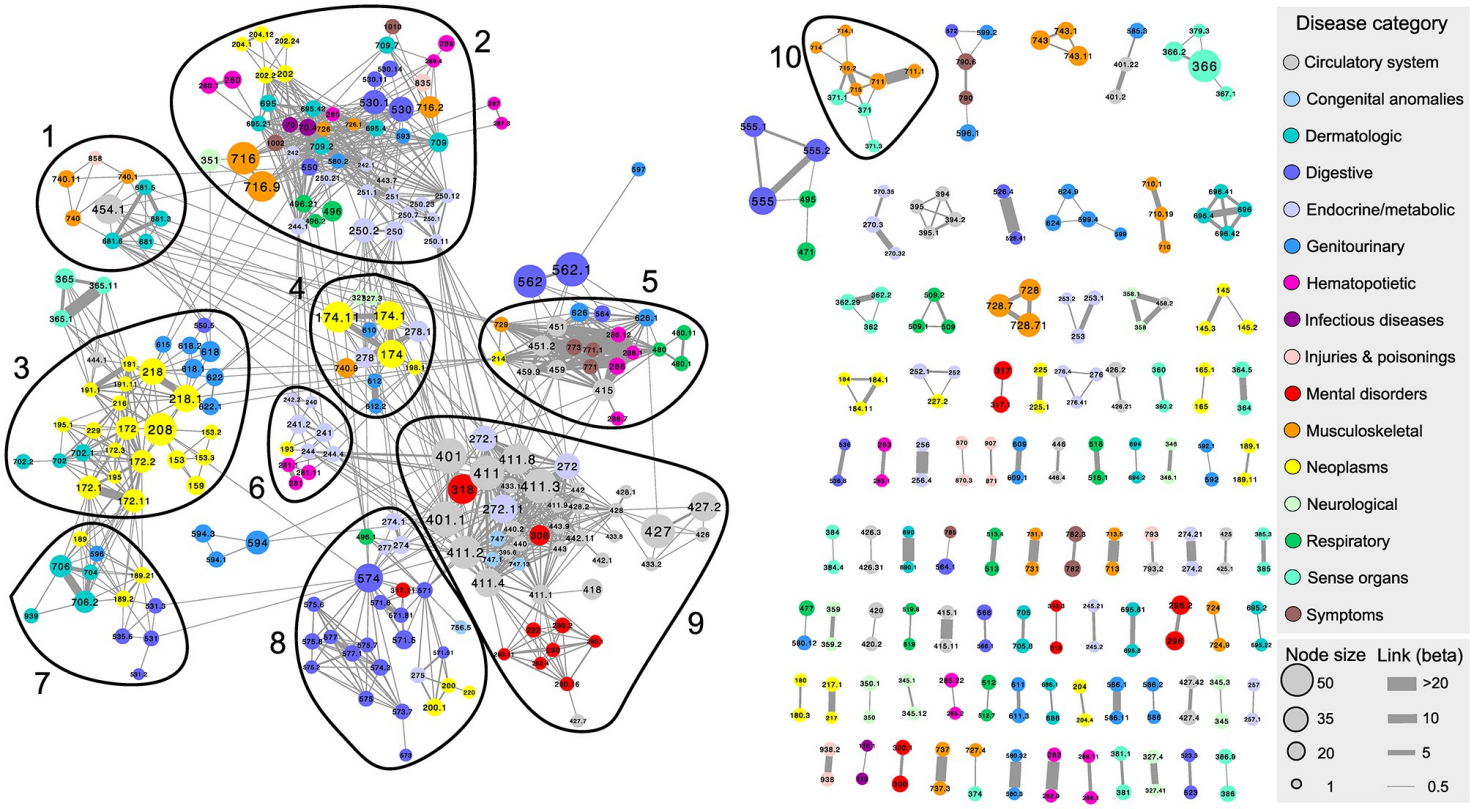
The UKBB based PheNet. The ten largest modules (size >5 nodes) are marked by circles, the node size corresponds to the number of SNPs associated to the disease and colored based on the disease category, as defined by the phenocode system. The link thickness corresponds to the link weight (*β*-score between the two diseases. Diseases without any links (singletons) are excluded from the network. A listing of modules and diseases is given in S2 File.

The UKBB based PheNet (Fig 2, S3 File) constructed from the updated list of SNP/LD-block-disease associations, where all included diseases are available in the HUNT study (7, 254 SNP-disease associations), includes 439 diseases with 1, 337 links between them. The full PheNet including all diseases with shared connections from the PheWAS summary statistics can be found in S2 Fig in S1 File. Most diseases are linked in a giant component and several smaller components, confirming that also at a genomic level, the genetic origin of many diseases are shared with other diseases. The nodes, representing diseases, are colored based on their disease category, sized by the number of associated SNPs/LD-blocks to the specific disease. A total of 2, 863 SNPs/LD-blocks are found in common for two or more diseases, representing 865 unique SNPs/LD-blocks. Most of the links (593) are based on specific SNPs in common, while the remaining are based on common LD-blocks. The link between diseases are represented by the *β*-scores and scaled accordingly in the figure. The ten largest modules identified by the Louvain clustering algorithm with the *β*-scores as link weights are circled in and numbered in the figure. Diseases with no connections to other diseases are excluded from the network and further network analysis. Most diseases are only connected by a few SNPs or LD-blocks, such as *Obesity* (phenocode 278.1) linked with *Essential hypertension* (phenocode 401.1), while links within hierarchical levels of the same phenocode (representing 22% of the number of links), such as *Benign neoplasm of uterus* (phenocode 281) linked with *Uterine leiomyoma* (phenocode 281.1) share up to 49 common SNPs or LD-blocks. This network consists of associations found from a solid framework for genomic association testing even with imbalanced case/control ratios, a large sample population for the association testing (UKBB participants), a stringent threshold for associations (*p*-value < 10^−6^), and adjustment for LD in linking diseases based on common SNPs, overcoming limitations of other studies [8–11, 14].

The number of diseases from each category in the PheNet and the mean degree within the category are shown in Table 1. All categories except pregnancy complications are represented, where the circulatory system (*n* = 58) and neoplasms (*n* = 55) are the disease categories with the most entries. Congenital anomalies (*n* = 4) and infectious diseases (*n* = 4) are the categories with the fewest diseases in the PheNet, but the ones with the highest mean degrees. Diseases of the circulatory system category are on average linked to 9.9 diseases, and most of them are located in module 9, which is dominated by diseases from the this category (see S2 File). Several cardiovascular diseases are known to be heritable, and many studies aim to understand the genetic causes of these diseases [29, 30]. In module 9, we find that *Coronary atherosclerosis* (phenocode 411.4) and *Myocardial infarction* (phenocode 411.2) are the diseases with the highest degree within the modules, and the link between these two diseases are also one of the strongest in the modules (*β* = 3.0). These diseases are closely related, sharing the same higher hierarchical phenocode level, and are caused by reduced blood flow to the heart. Previous studies have found several genetic markers prone to cause these diseases [31, 32], and it is interesting to see that they also linked to several other cardiovascular diseases, such as *Heart valve replaced* (phenocode 395.6) *Essential hypertension* (phenocode 401.1), and several endocrine/metabolic disorders and mental disorders.

**Table 1:** Number of diseases of each category for the PheNet and the HUNT sub-PheNet.

	PheNet		HUNT	
Category	*N*	Mean degree	*N*	Mean degree
circulatory system	58	9.9	50	7.4
congenital anomalies	4	12.3	3	12.0
dermatologic	34	6.8	30	2.7
digestive	42	6.1	38	3.6
endocrine/metabolic	47	8.7	42	4.0
genitourinary	38	3.8	34	2.1
hematopoietic	22	4.7	22	2.2
infectious diseases	4	10.5	2	1.0
injuries & poisonings	9	1.8	4	1.5
mental disorders	18	5.0	17	3.0
musculoskeletal	34	3.5	33	2.5
neoplasms	55	7.3	53	3.3
neurological	17	2.1	16	1.5
respiratory	21	3.5	20	2.3
sense organs	26	2.1	25	1.6
symptoms	10	7.0	9	2.3

### Neighbouring diseases tend to belong to the same disease category

Visually inspecting the PheNet in Fig 2, it may appear that diseases from the same category tend to cluster in the same network neighbourhood, i.e. that many nodes show homogeneity in the categories of their directly linked diseases. This is to be expected, since many of the diseases within a category are quite similar and, hence, might be genetically associated with the same diseases, as well as links within the hierarchical levels of the phenocodes. Examples of this strong assortativity are available in modules 3, 8 and 9, which mainly consist of diseases from the neoplasm, digestive and circulatory system categories, respectively, with a clear majority of direct links between diseases of the same category. In contrast, we can see that modules 2 and 5 consist of several disease categories, and there is no clear pattern in which categories are directly linked.

Thus, to systematically quantify the degree to which diseases from the same category are dominating their network neighborhood, we calculate the (homogeneity) *H*-score for each disease (node). For instance, we find that *Benign neoplasm of colon* (phenocode 208), the largest node in module 3 in Fig 2 has an *H*-score of 0.85: It is connected to 13 diseases, of which twelve belong to the same disease category, the neoplasms category. Also *Thyroid cancer* (phenocode 193), a neoplasms disease surrounded by only endocrine/metabolic disorders in module 6, has an *H*-score of unity. On the other hand, *Arthropathy NOS (phenocode 716.9)*, a musculoskeletal disease in module 6, has eleven neighbours, four of the musculoskeletal category and seven diseases of six different disease categories, giving a low *H*-score of 0.13. Hence, *Benign neoplasm of colon* and *Thyroid cancer* show high homogeneity of disease categories in their genetically defined neighbourhood where all diseases connected to them represent the same disease category, while *Arthropathy NOS* shows low homogeneity as it is genetically linked to diseases belonging to diverse categories.

To test if the diversity of neighbouring categories is less prominent in the PheNet than expected by chance, we first compare the mean *H*-score for all diseases in the PheNet to the mean *H*-score in 10^4^ random networks with the same network properties. In contrast to our network, where we expect that diseases from the same category cluster together, a random network should have an absence of pattern in the disease connections, resulting in low *H*-scores. Confirming our expectations, the mean *H*-score in the PheNet, ${\bar{H}}_{PheNet}=0.71$, is significantly larger than those in random networks (${\bar{H}}_{rand}=0.43$ with *p*-value < 10^−4^).

**Fig 3:**
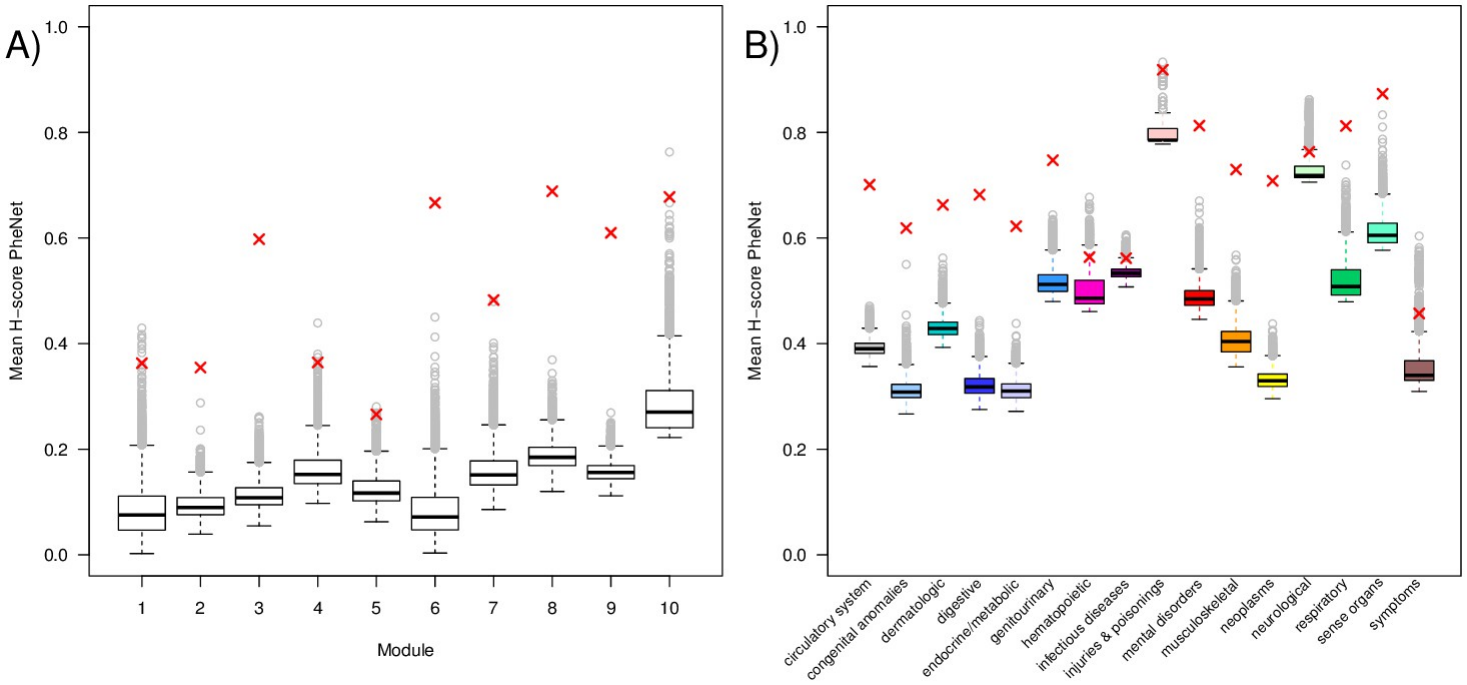
Mean H-score of phenotype network compared to 10^4^ random networks. Mean H score across the ten largest modules A) and across the 16 phenotype categories B). The red x-es shows the results from the PheNet, while the boxes with whiskers and outliers shows the results from 10^4^ simulated networks.

As we have seen above, the diversity of categories represented in each module varies. To further investigate the disease homogeneity on a module level, we calculate the mean *H*-score for each separate module and compare it with the corresponding results of the 10^4^ random networks. Fig 3A) shows the distribution of the mean module-*H*-scores of the random networks where the corresponding values from the PheNet are marked with a red X. We find that for all modules, the mean *H*-score of a module is significantly larger than expected by chance (see *p*-values in Table 2). Even though the *H*-scores for module 1, 2, 4 and 5, consisting of several phenocode categories, are lower than the other modules, they are still significantly larger than expected, i.e, the disease categories of the neighbourhoods are more homogeneous than expected by chance.

**Table 2:** Mean H-score with corresponding Bonferroni adjusted p-values for each module.

PheNet			HUNT		
Modules	Mean H	*p*-value	Modules	Mean H	*p*-value
1	0.36	< 10^−4^			
2	0.35	< 10^−4^	2*a*	0.47	< 10^−4^
			2*b*	0.65	< 10^−4^
			2*c*	0.54	< 10^−4^
3	0.60	< 10^−4^	3*a*	0.81	< 10^−4^
			3*b*	0.44	0.08
4	0.36	0.003	4	0.52	0.02
5	0.27	0.003	5	0.47	0.003
6	0.67	< 10^−4^	6	0.67	< 10^−4^
7	0.58	< 10^−4^	7	0.64	< 10^−4^
8	0.69	< 10^−4^	8	0.85	< 10^−4^
9	0.61	< 10^−4^	9*a*	0.75	< 10^−4^
			9*b*	0.82	< 10^−4^
10	0.68	0.001	10	0.62	0.03

We further investigate whether this homogeneity trend is present also for each separate disease category, as diseases from some categories might have more diverse network neighbourhoods than others. In Fig 3B) we see that the mean *H*-scores within most categories of the PheNet are significantly different from the random networks (*p*-values given in Table 3). We find that hematopoietic diseases, infectious diseases, injuries and poisonings, neurological diseases and the symptoms category all seem to be more diverse in their linked diseases. For symptoms, this makes perfectly sense, as the same set of symptoms might manifest with many diseases of quite different categories, and thus also share significant SNP-hits with co-occurring disease. Infectious diseases are only represented by four diseases in the PheNet, where two of them are located in module 2 and the other two are linked outside of the larger modules. *Chronic hepatitis* (phenocode 70.4) is one of the infectious diseases in module 2 and is linked to 25 other diseases of different categories. This could indicate the patients with *Chronic hepatitis* are genetically susceptible to many other types of diseases, such as *Obstructive chronic bronchitis* (phenocode 426.21) and *Hypoglycemia* (phenocode 251.1).

**Table 3:** Mean H-score with corresponding Bonferroni adjusted p-values for each category.

	PheNet		HUNT	
Category	Mean H	*p*-value	Mean H	*p*-value
circulatory system	0.70	< 10^−4^	0.79	< 10^−4^
congenital anomalies	0.62	< 10^−4^	0.70	< 10^−4^
dermatologic	0.66	< 10^−4^	0.88	< 10^−4^
digestive	0.68	< 10^−4^	0.85	< 10^−4^
endocrine/metabolic	0.62	< 10^−4^	0.77	< 10^−4^
genitourinary	0.75	< 10^−4^	0.82	< 10^−4^
hematopoietic	0.56	0.76	0.82	< 10^−4^
infectious diseases	0.56	0.40	1.00	1
injuries & poisonings	0.92	0.01	1.00	0.10
mental disorders	0.81	< 10^−4^	0.93	< 10^−4^
musculoskeletal	0.73	< 10^−4^	0.77	< 10^−4^
neoplasms	0.71	< 10^−4^	0.82	< 10^−4^
neurological	0.76	1	0.95	0.02
respiratory	0.81	< 10^−4^	0.87	< 10^−4^
sense organs	0.87	< 10^−4^	0.95	< 10^−4^
symptoms	0.46	0.68	0.48	1

In total, these results strongly support that most of the *H*-scores in the PheNet are larger than expected, thus demonstrating that a network of genetically linked diseases shows stronger homogeneity in the linked disease categories than expected by chance. Also, diseases with low *H*-scores, such as *Chronic hepatitis* and *Arthropathy*, could be interesting diseases to consider for further investigation of common genetic effects to other diseases.

### Linkage pattern between disease categories

**Fig 4:**
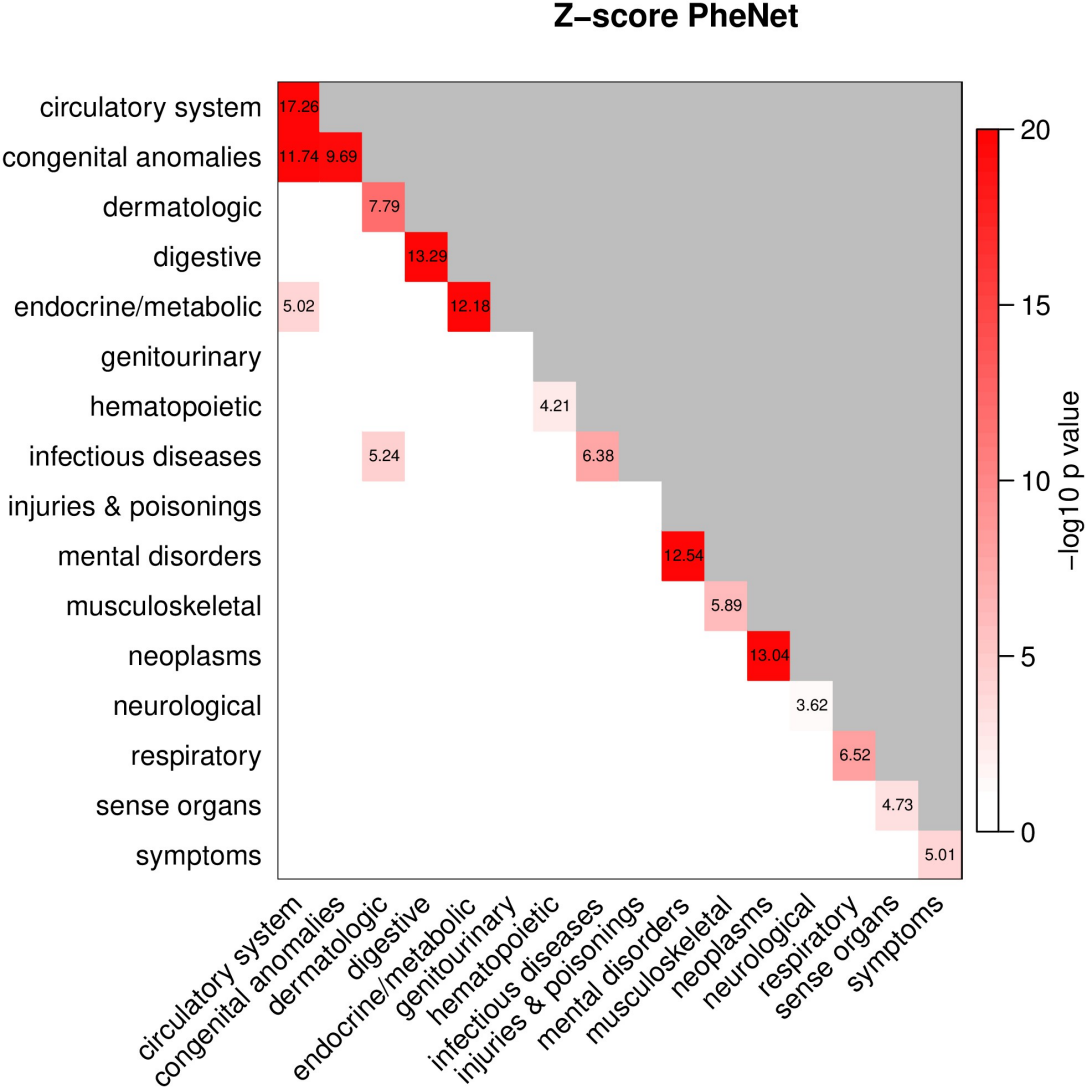
Z-score of overlap between categories. Entries show the Z-value where the corresponding two sided *p*-value adjusted for multiple testing (136 tests) have *p* < 0.05. These entries are colored based on the base-10 exponent of the *p*-value from the Z-test. Only Bonferroni significant associations are highlighted and shown with values.

Next, we investigate if some of the disease categories share a larger number of linkage than expected by chance. For this, we calculate the *Z*-score of the number of links connecting two categories, where the number of interactions between categories is standardized against 10^4^ random simulated networks. As already pointed out in the *H*-score analysis, many diseases of the same disease category tend to group together. The results from the *Z*-score analysis (shown in Fig 4) confirm that most of the categories display a significant number of direct intra-category connections. In addition, the circulatory system category has significantly elevated number of inter-category connections with the congenital anomalies and endocrine/metabolic categories, and the infectious diseases category has significant overlap with the dermatological diseases category. This result shows that diseases from these categories are significantly genetically linked and thus cluster in the network.

Congenital anomalies are represented by four diseases in the PheNet, where three of them, *Cardiac and circulatory congenital anomalies, Cardiac congenital anomalies and Congenital anomalies of great vessels* (phenocode 474, 474.1, 474.13), are linked to several circulatory system diseases in module 7. This result suggests that cardiac, circulatory and great vessels anomalies present at birth are genetically linked to other cardiovascular diseases appearing at a later state in life. The endocrine/metabolic disease category (colored light purple in Fig 2) is present in module 2, 4, 6, 8 and 9, and dominates the linking between these modules. This category includes several diseases for *Type I* and *Type II diabetes* (phenocode 250.1 and 250.2), which are known to be associated to several diseases, among them *Ischemic heart disease* and *Myocardial infarction* (phenocode 411 and 411.2) [33, 34] which we also observe in the PheNet. Module 2 holds two of the four infectious diseases, *Viral and chronic hepatitis* (phenocode 70 and 70.4), and they are highly linked to the dermatological and endocrine/metabolic diseases in this module.

### The HUNT sub-PheNet holds the same network properties as the PheNet

**Fig 5:**
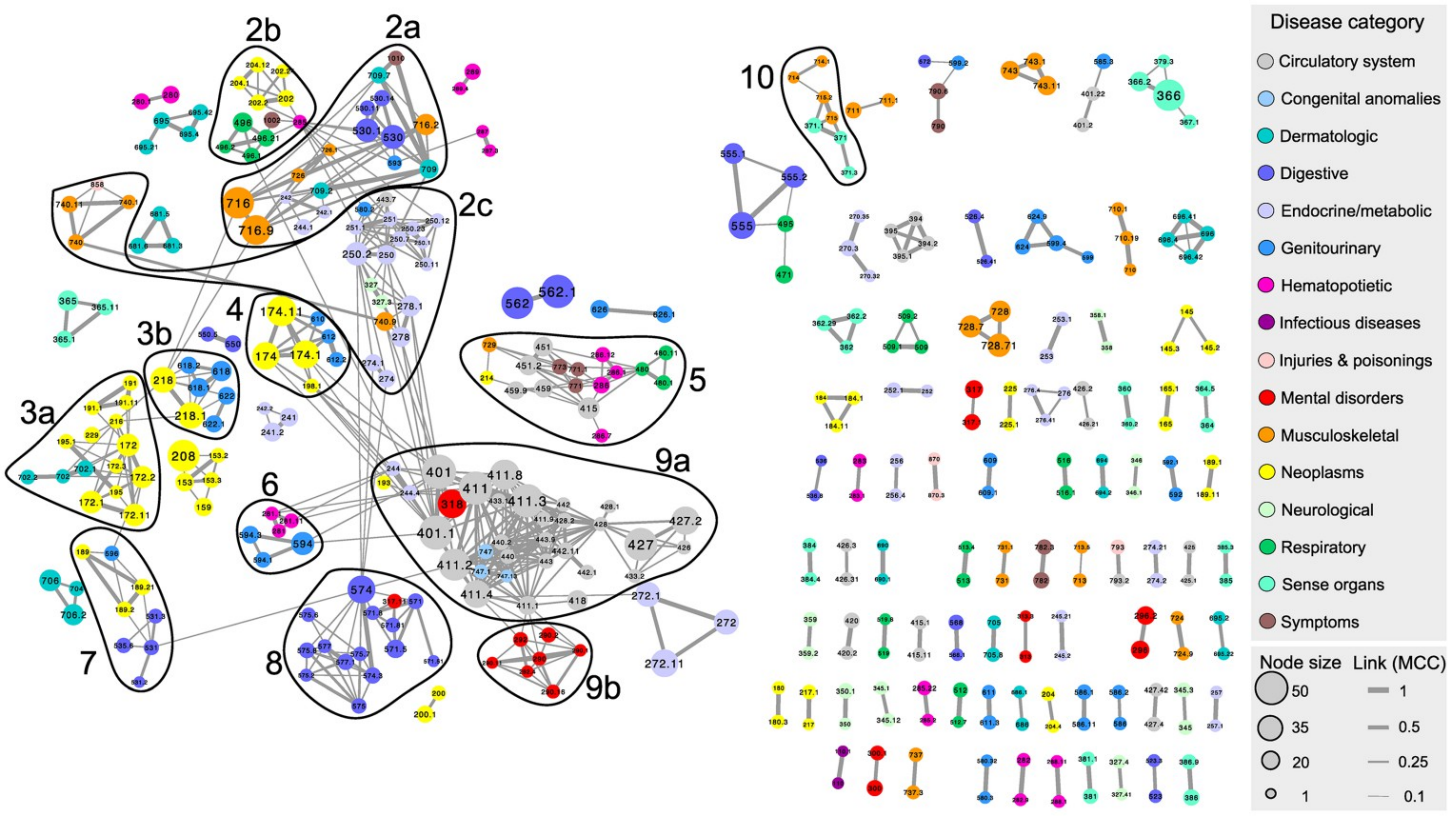
The HUNT sub-PheNet. The HUNT sub-PheNet is a sub-network of the PheNet where the only links kept are between diseases with a significant co-occurrence observed in the HUNT study. The figure features are mostly the same as for the PheNet. The link weight is represented by the MCC-score and diseases with no links to other diseases (singletons) have been removed. Modules identified for the HUNT sub-PheNet are marked.

**Fig 6:**
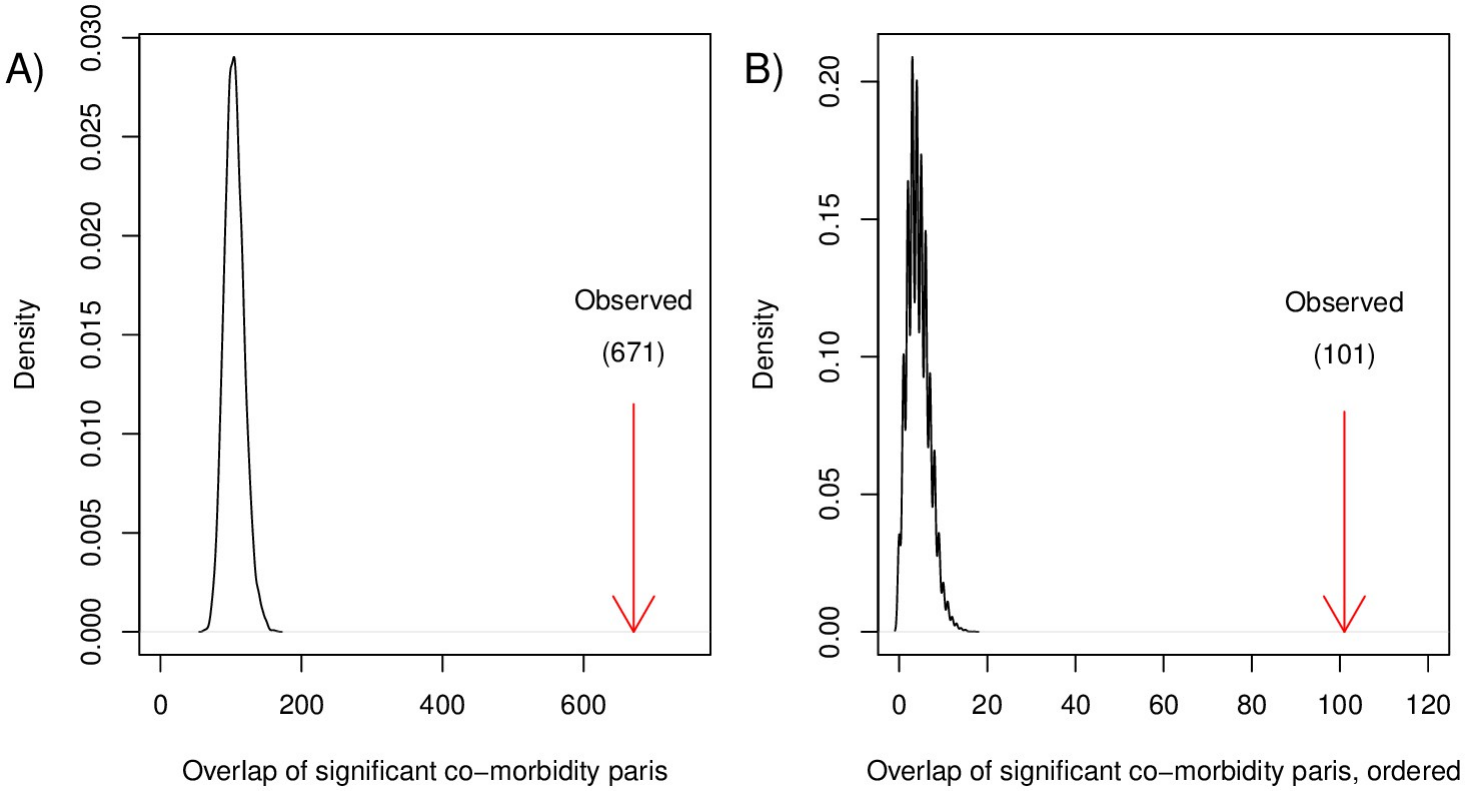
Overlap of significant unordered A) and ordered B) comorbidities. Distribution of the number of disease pairs with Bonferroni adjusted significant MCC-scores in the 10^4^ random networks. The observed number of significant disease pairs from the PheNet and HUNT sub-PheNet are marked with the red arrows.

Now that we have studied the PheNet of genetically linked diseases based on UKBB study participants and its properties, we seek to investigate if these network properties are maintained when considering only disease pairs that show strong co-occurrence in the HUNT study population. Since the sample size of the HUNT population is smaller than the UKBB population, we expect a smaller size of the HUNT sub-PheNet, where diseases with low prevalence are not represented by enough participants to identify strong comorbidities. Extracting the sub-network of the PheNet where disease pairs hold a Bonferroni adjusted significant MCC, the HUNT sub-PheNet shown in Fig 5 consists of 398 diseases with 671 links between them. This number of links is far more than expected based on simulated random networks holding the same network properties, where the mean number of significant co-occurrences is approx. 105, as shown in Fig 6A). The HUNT sub-PheNet is hence a network showing genetically linked diseases that also show strong co-occurrences in a different study population, where the network is far denser than expected by chance.

Also for the HUNT sub-PheNet, all disease categories except for pregnancy complications are represented, where the neoplasms and circulatory system categories are still the disease categories with the largest representation in the network, and congenital anomalies, infectious diseases and injuries and poisonings are the categories that are the least represented in the network, as shown in Table 1. The injuries and poisoning diseases and infectious diseases categories have lost five out of nine and two out of four diseases respectively due to low co-occurrences, while the other categories have lost smaller percentages of the number of diseases from the PheNet. This indicates that most genetically linked diseases of the other disease also show high co-occurrence, while the genetic disease links to the infectious diseases and injuries and poisoning categories show lower co-occurrence. As a consequence of the reduced network, the mean degrees within disease categories have also been reduced. Congenital anomalies with only three disease in the HUNT sub-network, still has the highest degree sharing links with 12 diseases, while the degree for diseases of infectious diseases has dropped to 1. The circulatory system diseases are still represented with high mean degree (7.4).

Even though the HUNT sub-PheNet is a sub-network and more sparse than the PheNet, the general structure of the network still holds when considering only pairs of diseases with strong co-occurrences. Using the MCCs as link weights, the Louvain clustering method identifies 13 modules in the HUNT sub-PheNet, displaying marked overlap with the 10 modules identified in the PheNet (see S3 Fig in S1 File). The difference of link strength in constructing these modules could potentially create non-overlapping modules, and the fact that we see much overlap between these modules indicates that the genetically linked diseases gives rice to the same module structure as the co-occurrence based modules. Module 2 from the PheNet has been split up into three modules, named 2*a*, 2*b* and 2*c* in the HUNT sub-PheNet, where module 2*c* also covers diseases from module 1 and 4 from the PheNet. Module 3 and 9 has also been split into two modules, 3*a*, 3*b* and 9*a*, 9*b*, in the HUNT sub-PheNet, where the genitourinary and neoplasms of uterus diseases from module 3 and the mental disorders from module 9 form own modules (3*b* and 9*b*) in the HUNT sub-PheNet.

Interestingly, in addition to the split of module 3 in the PheNet, the links between *Benign neoplasm of colon* (phenocode 281) and groups of *Brain, nervous system, skin and uterus neoplasms and cancers* (phenocodes 191, 172 and 218) do not show strong co-occurrence and are not linked in the HUNT sub-PheNet. This shows that even though these diseases are genetically linked to the other diseases of module 3 in the PheNet, they do not show significant comorbidity in a different study population. *Skin cancer* (phenocode 172) however is still linked to *Brain and nervous system cancer* (phenocode 191) and *Benign neoplasm of skin* (phenocode 216) is linked to *Benign neoplasm of uterus* (phenocode 218). Also, the mentioned *Chronic hepatitis* that is genetically linked to 25 diseases in the PheNet, indicating that patients with *Chronic hepatitis* are genetically susceptible to these 25 other diseases, are not included in the HUNT sub-PheNet. It might be that these genetic links are still valid, but they do not show strong co-occurrences in the HUNT study-population either because they are not valid or due to lower sample size of the HUNT population.

Performing the *H*-score analysis on the HUNT sub-PheNet, we find that the mean *H*-score for all the diseases in the network, ${\bar{H}}_{HUNT}=0.83$, is significantly larger than expected in random networks (*H*_*rand*_ = 0.52 with *p*-value < 10^−4^), and the mean *H*-score for most modules and categories are significantly larger than expected (see Tables 2 and 3, and S4 Fig in S1 File for further details). These results demonstrate that, reducing the genetically linked disease network to only include links showing strong co-occurrences, the disease connections are kept at an even larger degree within the same disease categories.

To point to some of the differences between the two networks, we find that module 3*b* is the only module with a non-significant mean *H*-score in the HUNT sub-PheNet. For disease categories, we find the same pattern in the HUNT sub-PheNet as for the PheNet in 13 of the 16 disease categories. The difference is limited to the hematopoietic and neurological categories that show significant mean *H*-scores in the HUNT sub-PheNet, and the injuries and poisonings category, which shows a non-significant mean *H*-score in the HUNT sub-PheNet while it is opposite for these categories in the PheNet. Diseases from the hematopoietic and neurological categories have hence reduced the links to other disease categories and becoming more homogeneous in their neighbourhood, while the opposite is observed for diseases from the injuries and poisonings category.

The results for the *Z*-score analysis of the HUNT sub-PheNet (see S5 Fig in S1 File) differ in four category pairs from the corresponding analysis of the PheNet, while the remaining 132 category pairs show similar results. Thus, when reducing the genetically based disease network to only include those with also strong co-occurring diseases, the significance of interactions across categories are mainly maintained. The four differences are as follows: Infectious diseases are only represented by two diseases in the HUNT sub-PheNet (Chronic and Viral hepatitis), which are only connected to each other, so the significant number of interactions between this category and dermatological diseases from the PheNet is not available in the HUNT sub-PheNet. Also, both the genitourinary and injuries and poisonings categories show significant overlap with themselves in the HUNT sub-PheNet. Interestingly, the significant number of interactions between the circulatory system and endocrine/metabolic disease categories from the PheNet is no longer significant in the HUNT sub-PheNet. Inspecting the PheNet in Fig 2, we found several links between the *Type 1 diabetes with ketoacidosis* (phenocode 250.11) in module 2 and the circulatory system diseases in module 7, while in the HUNT sub-PheNet these links have been removed due to weak co-occurrence. As a consequence, the resulting number of interactions between these categories is no longer significant.

To summarize, we find that the network properties in the genetically linked PheNet and the genetically and strong comorbidity linked HUNT sub-PheNet are mostly maintained. However, the differences pointed out in this section highlight the importance of validating the genetically linked diseases with co-occurrence data in a different study population. Even though many diseases are genetically linked, they may not display strong co-occurrence in a different study population, indicating that a person genetically predisposed for both diseases not necessarily will have an increased chance of obtaining both. More importantly, these networks demonstrate links between diseases that are genetically linked and at the same time show high co-occurrences.

### Many disease pairs show strong ordering in disease history

In the HUNT study population, we also have access to information regarding *when* diseases occurred for each individual. Considering the date-ordered pairs of co-occurring diseases in the HUNT study population, we find that 161 of the pairs (taking into account both directions) show significant comorbidity, which is far more than expected based on simulations from random networks holding the same properties, see Fig 6B). This means that a large fraction of the disease pairs observed in the PheNet are actually observed in a specific time-sequence in the HUNT study population.

Following this observation, we investigate the temporal ordering of these disease pairs and question whether having one disease from the genetically linked and strong co-occurring diseases pairs in the HUNT sub-PheNet is associated with an increased chance of obtaining the other, independent of age and gender, and if this temporal ordering of the disease pair represents the major directional flow and is of significant frequency. Performing regression models for both directions of all disease pairs in the HUNT sub-PheNet that are not higher or lower level of the same phenocode (193 diseases with 437 links), we identify disease pairs where the contraction of one disease gives significantly higher odds of obtaining the other, when adjusting for age and gender of the patients. Further, performing binomial tests to ensure the significance of frequency of these directions and identifying the major temporal flow, we find 107 directed disease pairs between 71 diseases. Hence, roughly a fourth of these disease pairs are significantly observed in the specific time-sequence, where trivial ordering due to gender differences or different age of onset are adjusted for.

**Fig 7:**
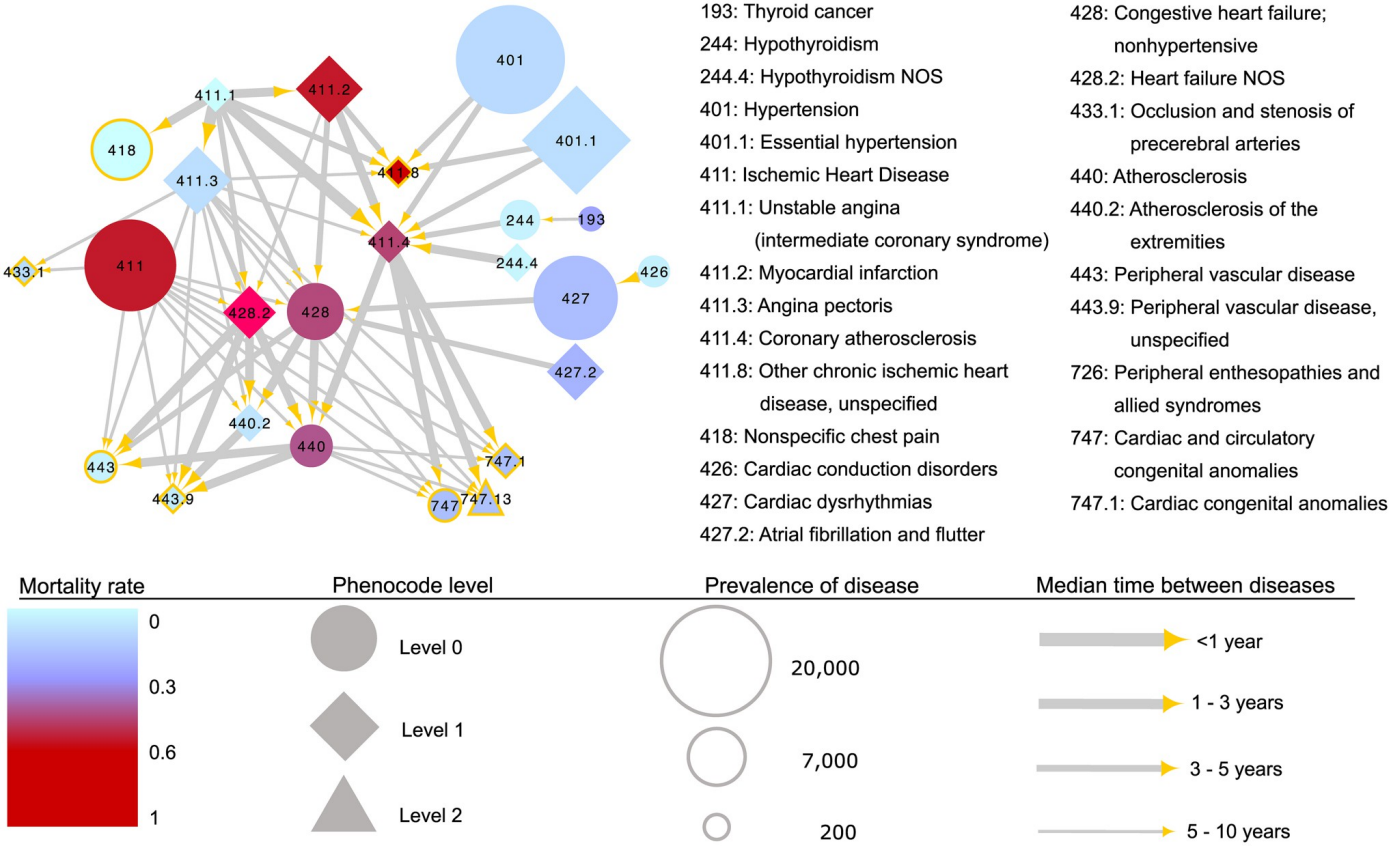
Network of ordered pairs. The giant component of ordered pairs of diseases where the arrows show the directions of the disease histories, scaled by the median time between the diagnosis. The nodes are scaled based on the prevalence, shaped according to the phenocode level and colored according to the mortality rate of the disease. Sink nodes are marked with yellow borders.

This directed disease network showing the temporal majority flow of significantly subsequent diseases consists of one giant component and 8 smaller isolated groups with variable sizes. The giant component shown in Fig 7 holds 60 links between 25 diseases, all from module 9*a* in the HUNT sub-PheNet, consisting of mostly circulatory system diseases and some from the congenital anomalies, endocrine and metabolic, and neoplasm disease category. In this figure, the size of the disease-nodes is scaled according to their prevalence in HUNT population (excluding patients with less than two years observation time), colored according to their mortality rate based on the Norwegian Cause of Death registry and shaped according to the phenocode level. Sink diseases (those without any subsequent diseases) are marked with a yellow border. The link thickness reflects the median time between events (with thicker corresponding to shorter time). Note that links between higher or lower levels of the same phenocode is not included, as all patients diagnosed with a level 1 or 2 phenocode is by definition also diagnosed with the higher level phenocode(s) at the same event.

The 25 diseases shown in Fig 7 are different levels of ten level 0-phenocodes. *Ischemic Heart Disease* (411) is represented by six phenocodes (411, 411.1, 411.2, 411.3, 411.4, and 411.8), where all are different specified versions of *Ischemic Heart Disease* with different prevalence and mortality rates. *Other chronic ischemic heart disease, unspecified* (phenocode 411.8), which has one of the highest mortality rates, is a sink node subsequent of *Myocardial infarction* (phenocode 411.2), *Unstable angina (intermediate coronary syndrome)* (phenocode 411.1), *Angina pectoris* (phenocode 411.3), *Hypertension* and *Essential Hypertension* (phenocde 401, 401.1). *Hypertension* and *Essential Hypertension*, which have high prevalence in the HUNT study population (∼22%) are major modifiable risk factors for cardiovascular disease. Even though they are genetically linked and shows strong co-occurrence with many cardiovascular diseases in the HUNT sub-PheNet, they are only preceding disease for *Coronary atherosclerosis* (phenocode 411.4) and *Other chronic ischemic heart disease, unspecified* in this directed network. Hence, there is not enough evidence to claim that the odds of obtaining, for example all forms of *Ischemic Heart Disease*, increase independently of age and gender when having hypertension. This is despite the fact that, as our results show, these diseases are linked both genetically and comorbidity-wise.

Additionally, we find that *Angina Pectoris* and *Myocardial infarction* are prevalent diseases both subsequent to *Unstable angina (intermediate coronary syndrome)*. They are both preceding of *Congestive heart failure; nonhypertensive* (phenocode 428), *Heart failure NOS* (phenocode 428.2), *Coronary atherosclerosis* and *Other chronic ischemic heart disease, unspecified*, which all have high death rates. *Myocardial infarction* has a higher death rate and also shorter time until these following diseases than *Angina Pectoris*, indicating that those surviving *Myocardial infarction* are of high risk of obtaining these other relative fatal diseases within few years.

Other interesting links in this network are the links of *Hypothyroidism* and *Hypothyroidism NOS* (phenocode 244 and 244.4) preceding *Coronary atherosclerosis*. Though it is known that hypothyroidism is a risk factor for heart conditions [35], we here find evidence for these diseases being genetically linked, have high co-occurrence in a different study population and are subsequent of each other independent of age and gender.

## Discussion

Human disease networks offer a potential road map for both clinicians and researchers studying various forms of diseases, uncovering how they are related on a large scale. While it is well known that environmental factors significantly affect the human immune system, and thus disease etiology [36], we have focused this work on understanding the interplay between disease and genetic factors. Previous studies have successfully created human disease networks of genetically linked disorders, either based on diseases linked through common genes [7] or genetic information [8–14]. Others have constructed disease networks entirely based on EHR-data with co-occurring diseases [24, 37]. Here, we combine the two approaches, creating a disease network based on genetic linkage and extract the sub network of EHR-based co-occurring diseases from a different study population. With this, we show that many of the genetically linked diseases are in fact co-occurring diseases, where we overcome limitations of bias towards much studied diseases, small sample sizes, different populations for the genetic studies, short follow-up of participants, LD-correlation, case/control imbalances and relatedness.

We are not the first to consider comorbidity among diseases based on genetically linked disease networks. Menche et. al. [38] created the interactome; genes found from OMIM and GWAS (genes with GWAS significant SNPs) linked through molecular interactions, and they showed that diseases with overlapping disease modules (overlapping genes associated to both diseases) show higher comorbidity than diseases without overlapping disease modules. However, they point out that the interactome is far from complete and that it is biased towards much studied diseases. Park et. al. [39] focus on the overlap between diseases linked though common genes from the HDN [7] and comorbidities based on EHR-based disease histories from U.S. Medicare [24]. They show that diseases linked through common genes show higher comorbidity, where particularly diseases linked through domain-sharing genes show higher comorbidity than diseases with non-domain-sharing genes. This indicates that using SNP-linked diseases rather than gene-linked diseases could be beneficial for the study of comorbidity.

While Dong et. al. [13] and Sriram et. al [14] both compared SNP-based disease interactions with observed comorbidities, they are limited by using the same study population for both genetic testing and overlap with comorbidities. Menche et. al. [38] and Park et. al. [39] are both biased towards much studied diseases and limited by noise from translating OMIM diseases to EHR-based diseases, where the disease annotations have different nomenclatures. Their comorbidity data are strong in number of participants, but limited in the time span. The U.S. Medicare EHR-data covers only four years of disease histories for elderly patients, likely resulting in many uncovered disease co-occurrences. In our work, we do not share the strength of roughly 13 million patients, instead the HUNT study is unique in covering up to 30 years of EHR-data for roughly 90, 000 patients. With this, we argue that our network presents an unbiased and more specific disease network, and that we uncover more of the co-occurring diseases when linking our SNP-based PheNet with EHR-based HUNT comorbidities.

Using SNP associations with diseases rather than genes, we do not consider if the SNP affects a gene that is causal for the disease. Grouping some SNPs into LD-blocks increases the genomic region for the genomic association, but this is only to catch situations where different SNPs are marked as top SNPs (lowest *p*-value) but are in fact in high LD and represent the same loci association to the disease. Some gene-disease associations might be excluded as different mutations in the gene and influence the expression of the gene, thus causing the disease. As an example, mutations in the BRCA1 and BRCA2 genes are well known to be associated with increased risk of breast cancer. However, several mutations exist, and they seem to be population specific [40]. According the the GWAS catalog [2], no mutations in BRCA1 and only a few mutations for BRCA2 are found to be GWAS significant.

However, basing our PheNet on SNP associations, we know exactly which change in a genetic position that is associated with the disease. More than 90% of the trait-associated variants detected though GWAS are located in non-coding sequences [41], and thus, excluding all variants not coding for genes or proteins reduces the ability to study genetic causes of diseases. With current methods for functional genome annotations, one can explore the functional consequences of both coding and non-coding sequence variants detected though GWAS and PheWAS [42]. Thus, genetic disease connections found in the PheNet could be used for targeted studies of functional implications of the SNPs and convergence to possible meaningful pathways, despite the fact that the SNPs are located in non-coding regions. However, as for any GWAS or PheWAS, the identifiable results are limited by the fact that only one SNP is tested against the disease at a time. For further understanding of the development and genetic architecture of diseases, interactions between environmental factors, epigenetic changes, and the presence of genetic factors are needed.

Even though we argue that the networks created in this study are based on more robust methods than the works cited above, there are also some limitations. First of all, the genetically linked diseases from UKBB are purely based on white British participants of European ancestry. Hence, we cannot conclude that the diseases are genetically linked for persons with different ancestry. This is a general problem in large population genomic studies, as most are conducted with people of European ancestry. As a consequence, this raises concerns about the utility of the health related outcomes from these genomic studies to patients of other ancestries and geographical locations [43].

Second, as we aimed to include as many strong disease links as possible from the PheWAS data, we used no threshold for the number of participants diagnosed with a disease. Some diseases might be linked where the prevalence of one or both diseases are low. Checking for strong co-occurrences in the HUNT population with relatively smaller sample size than the UKBB population, low prevalent diseases might not be represented enough to identify strong comorbidities. A larger sample size for the comorbidity identification could potentially identify more of these comorbidities in the PheNet. Additionally, the *p*-value threshold and MAF cutoff value are respectively set to 10^−6^ and 0.1%, which is less stringent than the generally excepted GWAS threshold of 5 ⋅ 10^−8^ and the often used MAF threshold of 1%. This choice potentially links diseases that would not have been linked with a standard GWAS significance threshold and MAF cutoff. Compared with other SNP-based disease networks, the *p*-value threshold use here is more stringent and the MAF less stringent [11, 13, 14, 44]. The MAF threshold should be set based on the size of the underlying population, and as the SNP-disease associations used here are based on the large UKBB population, we have used a MAF cutoff of 0.1%. As the main goal of this work is to identify which of the genetically linked disorders that are observed to co-occur, we argue that the questionable links will either be confirmed or suppressed when considering only the pairs of significantly co-occurring diseases from the HUNT study.

Third, even though the HUNT data are strong in its participation rate and have been ongoing for a very long time period, the registries used for this work are limited to ∼75% of the phenocodes from the UKBB. Also, not all of the health records span the entire HUNT study time frame (1987–2017). Apart from this fact our data covers hospital records for up to 30 years, which is a great advantage of our work.

Fourth, many of the diseases in our networks are quite similar. As an example, there are nine diseases corresponding to different types of diabetes. This is due to the inclusion of 2-digit phenocodes (3 levels). Using a higher level of the phenocode hierarchy could reduce the number of similar diseases, but would loose the specific information given by the PheNet. As an example, *Alzheimer’s disease* is a level 2 phenocode (290.11) with higher levels *Dementia* (290.1) and *Delirium dementia and amnestic and other cognitive disorders* (290). These are all linked to *Coronary atherosclerosis* (phenocode 411.4) through common identified SNPs. None of them are, however, linked to the level 0 disease *Ischemic heart disease* (phenocode 411), as no SNPs identified in the PheWAS were found in common for these diseases. In this example, merging phenocodes into 1-level associations (290.1–411.4) would loose information about the link between *Alzheimer’s disease* and *Coronary atherosclerosis*, and merging into a 0-level association (290–411) would in fact be incorrect, as there are no common SNPs associated with the broad terms *Delirium dementia and amnestic and other cognitive disorders* and *Ischemic heart disease*. Hence, keeping all phenocode levels in the PheNet provides detailed information about the associations, but with the downside of interpretation.

The EHR-based GWAS and PheWAS using phenocodes are also limited by the ICD-coding and phenocode systems. Finding a balance between having a specific enough definition and a definition that identifies enough cases is challenging, and some phenocodes are therefore poorly covered. Also, there might be variations in which diseases are observed and registered as ICD-codes, both in terms of the patient visits and the doctor’s ability to detect and register the diagnose. A better classification system for the diseases other than the phenocodes could be beneficial for a clearer disease network. Also, a few phenocodes cover the same ICD-codes, which potentially could link diseases simply because they are observed to be the same diagnosis code. Mapping to diseases in the Human Phenotype Ontology (HPO) [45] could overcome some of these limitations and provide useful information of shared hierarchical annotation structure of the disease features. With the phenocode, we restrict the network analysis to 16 disease categories. Using HPO, overlap between annotations could instead be applied. The HPO is however based on OMIN diseases and, as we in this work are restricted to summary statistics of phenocodes, the mapping from phenocodes to OMIN would involve mapping back to ICD-codes. This would cause both difficulties in determining which of the ICD-codes that would be assigned the phenocode-SNP association, in addition to noise when translating ICD-codes to OMIN-diseases (as we have seen above as a limitation in Ref. [38, 39]).

Finally, as for any GWAS or PheWAS, validation of the results in a separate population increases the confidence in the genomic findings. An even more robust disease network would have been one where the genetically linked diseases are validated in a separate population or with meta-analyses before extracting strongly co-occurring links. The co-occurring links could also be validated in yet another population. For the utility of this disease network in all populations, one could create disease networks based on genetic findings for all ancestries and genders, and due to genetic differences, one could also create ancestrally specific disease networks. We believe that the work presented here is a step towards more accurate precision medicine that, with future studies, might be beneficial for health wellness all around the world.

## Conclusion

In this study, we have created a network of genetically linked diseases that also show strong co-occurrence within a different study population. Our results show that many genetically linked diseases are observable as actual comorbidities, while some are genetically linked but do not appear to be comorbidities. The methods for creating the PheNet overcome limitations from previous studies, and we argue that the diseases linked in this network are based on more solid methods and datasets, and hence, being more reliable. We find that the number of overlapping disease pairs is far larger than expected by chance, and that the network properties are mostly maintained between the network of only genetically linked diseases and the network of both genetically linked and strongly co-occurring diseases. Many diseases are connected in larger components, and all disease categories, except for pregnancy complications, are included in the networks. Most diseases tend to link to other diseases of the same category, where some categories are more linked to each other than expected by chance. We have also extracted a directed network of consecutively occurring diseases, where these diseases also show high co-occurrence and are genetically linked. Being diagnosed with the first disease significantly increases the odds of obtaining the next, independent of age and gender, and these disease pairs show significant frequent pattern in the temporal ordering. The majority temporal trajectories network displays a giant component consisting of mostly cardiovascular diseases, where diseases with high mortality rates are often proceeding of other less severe diseases, and with shorter time until their next diseases.

We argue that the work presented here could be of great benefit to researchers and clinicians and used as a resource to study and explain relationships between diseases. Potential uses cases include studying how two or more diseases of interest are located in the networks, if they are directly linked and then by which common genetic variant(s), if they are linked through a common disease, and then how this common disease is genetically and co-morbidity-wise linked to them, or using the ordered disease network to study if these diseases are subsequent to each other. Hopefully, this is a step towards more accurate precision medicine, and we hope that further improved networks for diverse ancestries could be beneficial to not only the European ancestral populations, but to people of all ancestries and genders.

## Supporting information

S1 File Supporting figures showing additional network properties. S1: Distribution of ages and birth year for the 90, 103 HUNT study participants registered at hospital or general practitioner visits. A) Distribution of ages at first and B) last observed visit. C) Distribution of the time frame for each participants observed visits. D) Distribution of the birth year of the participants. S2: The full PheNet not reduced to include only diseases observed in the HUNT study. S3: Overlap between diseases in modules of the PheNet and the HUNT sub-PheNet. The colorbar shows the base-10 exponent of the *p*-value for the overlap. S4: Mean H-score of HUNT sub-PheNet compared to 10^4^ random networks. Mean H score across the 13 largest modules A) and across the 16 phenotype categories B). The red x-es shows the results from the HUNT sub-PheNet, while the boxes with whiskers and outliers shows the results from 10^4^ simulated networks. S5: Z-score of overlap between categories in the HUNT sub-PheNet. Entries show the Z-value where the corresponding two sided *p*-value adjusted for multiple testing (136 tests) have *p* < 0.05. These entries are colored based on the base-10 exponent of the *p*-value from the Z-test. Only entries with Bonferroni significant associations are highlighted and shown with numbers. S6: The full network of ordered pairs of diseases where the arrows show the directions of the disease histories, scaled by the median time between the diagnosis. The size, shape and color of the nodes represents the prevalence, phenocode level and mortality rate of the disease.
(PDF)

S2 File List of diseases in the PheNet. List of diseases in the PheNet with their phenocodes names displayed in the networks, disease categories, in which module they are located in the PheNet and in the HUNT sub-PheNet and the *H*-score in the PheNet and HUNT sub-PheNet.
(TXT)

S3 File Edgelist for the PheNet. An edgelist containing the disease pairs linked in the PheNet with columns From/To: the diseases, N: number of shared SNPs/LD-blocks, betaMerged: the *β*-score of the link, FromCode/ToCode: phenocode of the diseases, SNPs: which SNPs that are shared between the diseases.
(TXT)

S4 File Edgelist for the HUNT sub-PheNet. An edgelist containing disease pairs linked in the HUNT sub-PheNet, with columns From/To: the diseases, N: number of shared SNPs/LD-blocks, betaMerged: the *β*-score of the link, FromCode/ToCode: phenocode of the diseases, C: number of participants with both diseases, MCC: the MCC-score of the link, and fisherp: the *p*-value of the Fisher’s exact test (showing only links from S3 File where this value is significant).
(TXT)

S5 File List of diseases in the directed network. List of diseases in the directed network with columns Phenocode: phenocode of disease, Disease: disease name, Prev: prevalence in HUNT, Dprev: mortality rate in HUNT (number of death due to this disease divided by the prevalence, note that one disease has mortality rate >1, as a result of participants dying of this disease before a registered visit to the hospital or general practitioner), Category: phenocode category, Sink: (0/1) if the disease is a sink node, and Level: the phenocode level of the disease.
(TXT)

S6 File Edgelist for the directed network. An edgelist containing the directed disease pairs from the network of ordered pairs with columns First/Second: phenocodes of diseases, with first meaning before the other, MedianTime: median time between these diseases for participants with this ordering, and Time: categorical variable for the median time (1: < 1*y*, 2: 1 − 3*y*, 3: 3 − 5*y*, 4: 5 − 10*y*, 5: >10*y*).
(TXT)
