# Supplementary material for: Phenome-wide association network demonstrates close connection with individual disease trajectories from the HUNT study
Source: PLoS One. 2024 Dec 27;19(12):e0311485. doi: 10.1371/journal.pone.0311485 (PMC11676826; doi:10.1371/journal.pone.0311485)
Supplement: S1 File — S1: Distribution of ages and birth year for the 90, 103 HUNT study participants registered at hospital or general practitioner visits. A) Distribution of ages at first and B) last observed visit. C) Distribution of the time frame for each participants observed visits. D) Distribution of the birth year of the participants. S2: The full PheNet not reduced to include only diseases observed in the HUNT study. S3: Overlap between diseases in modules of the PheNet and the HUNT sub-PheNet. The colorbar shows the base-10 exponent of the p-value for the overlap. S4: Mean H-score of HUNT sub-PheNet compared to 104 random networks. Mean H score across the 13 largest modules A) and across the 16 phenotype categories B). The red x-es shows the results from the HUNT sub-PheNet, while the boxes with whiskers and outliers shows the results from 104 simulated networks. S5: Z-score of overlap between categories in the HUNT sub-PheNet. Entries show the Z-value where the corresponding two sided p-value adjusted for multiple testing (136 tests) have p < 0.05. These entries are colored based on the base-10 exponent of the p-value from the Z-test. Only entries with Bonferroni significant associations are highlighted and shown with numbers. S6: The full network of ordered pairs of diseases where the arrows show the directions of the disease histories, scaled by the median time between the diagnosis. The size, shape and color of the nodes represents the prevalence, phenocode level and mortality rate of the disease. (PDF) [file pone.0311485.s001.pdf]

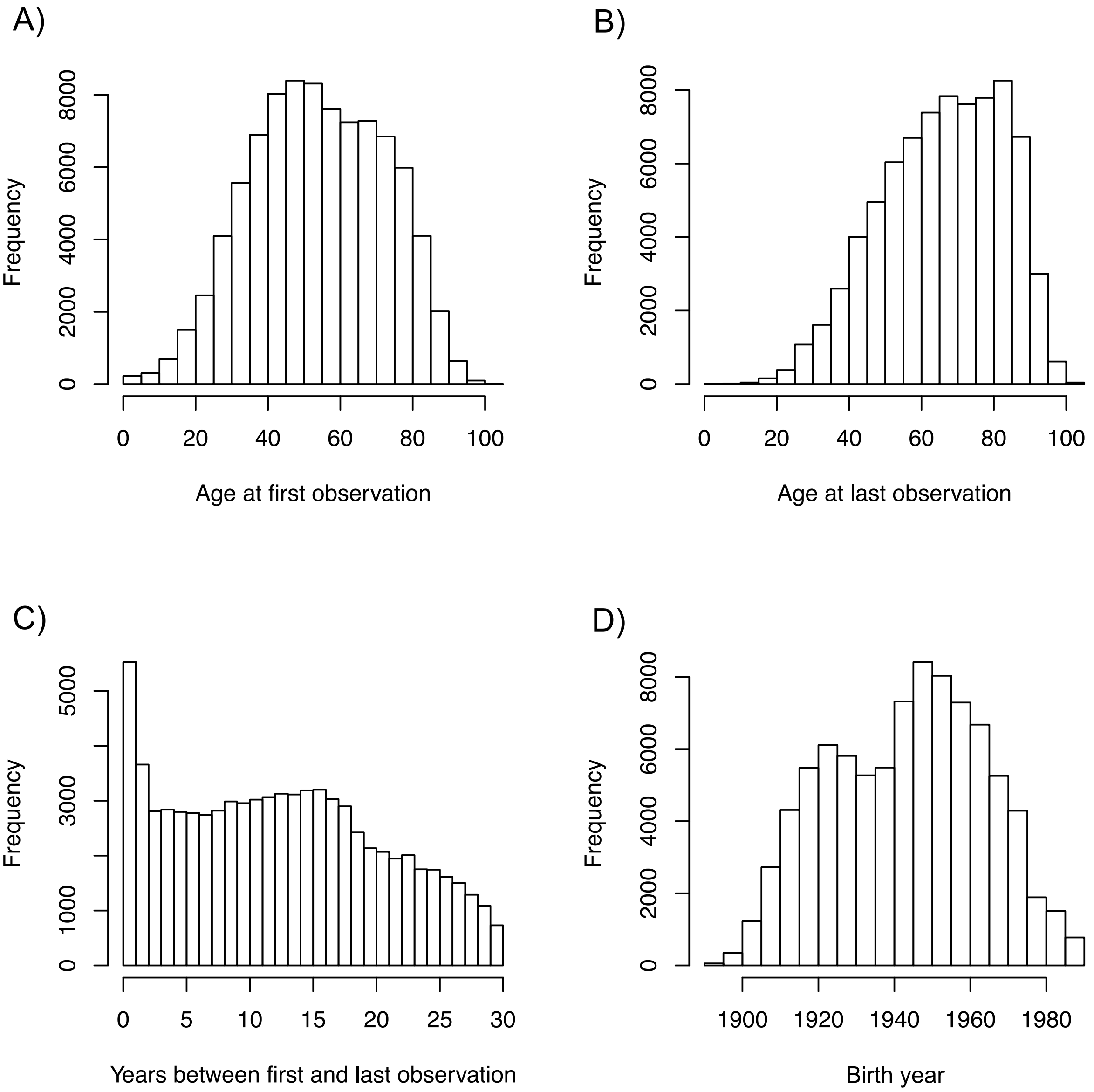

Fig. S1: Distribution of ages and birth year for the $90,103$ HUNT study participants registered at hospital or general practitioner visits. A) Distribution of ages at first and B) last observed visit. C) Distribution of the time frame for each participants observed visits. D) Distribution of the birth year of the participants.

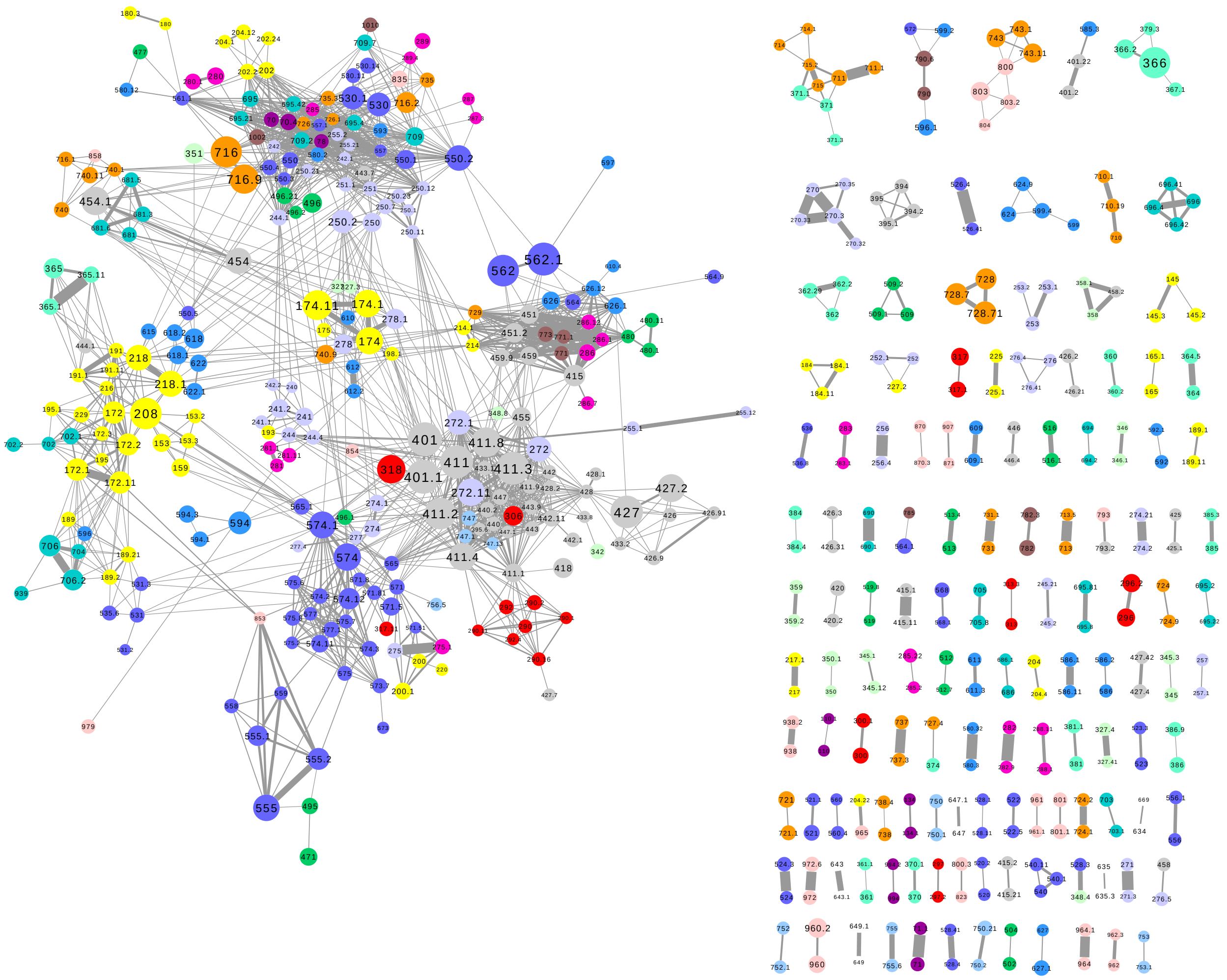

Fig. S2: The complete UKBB PheNet, not reduced to include only diseases observed in the HUNT study.

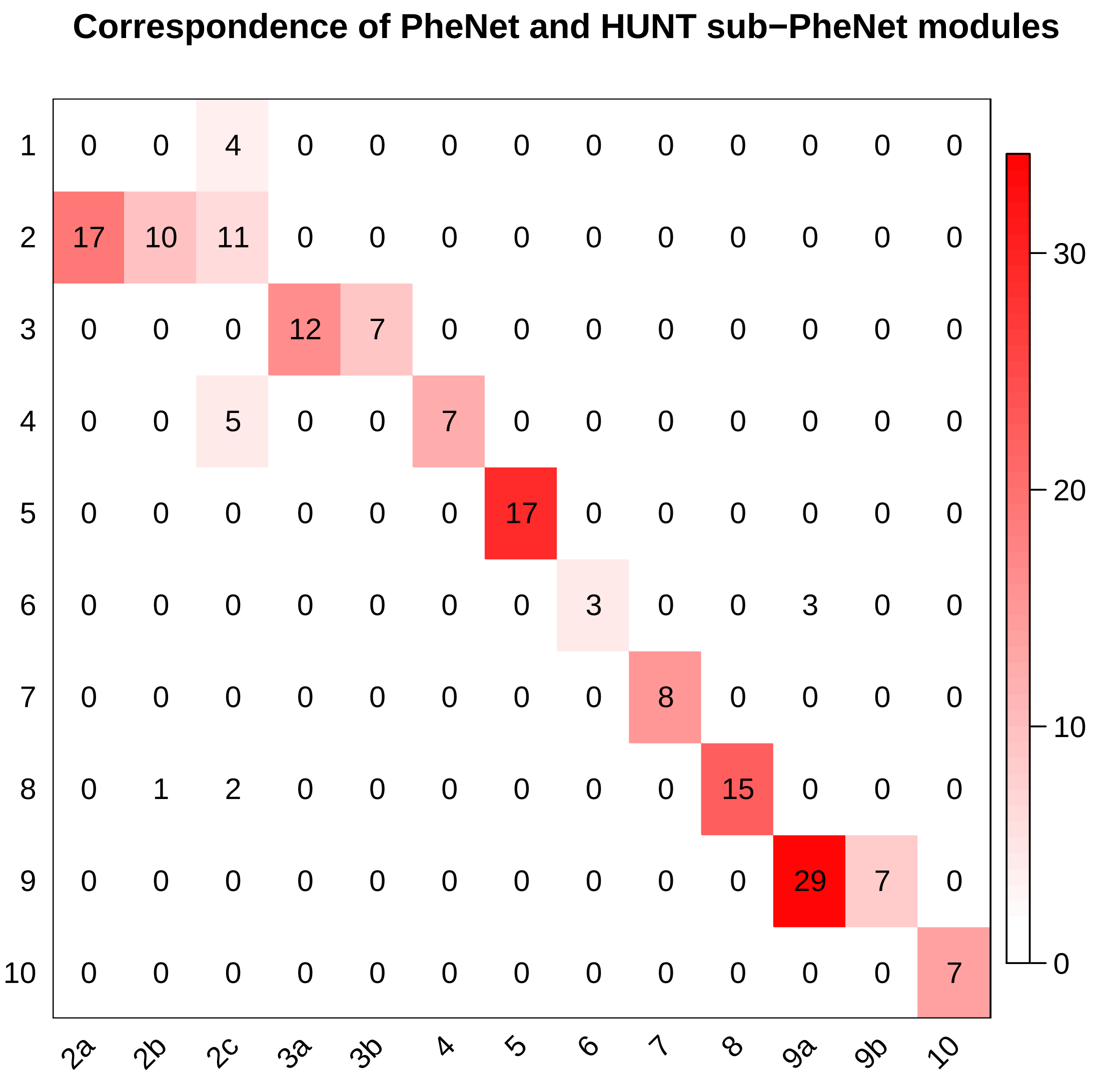

Fig. S3: Overlap between diseases in modules of the PheNet and the HUNT sub-PheNet. The colorbar shows the base-10 exponent of the p-value for the overlap.

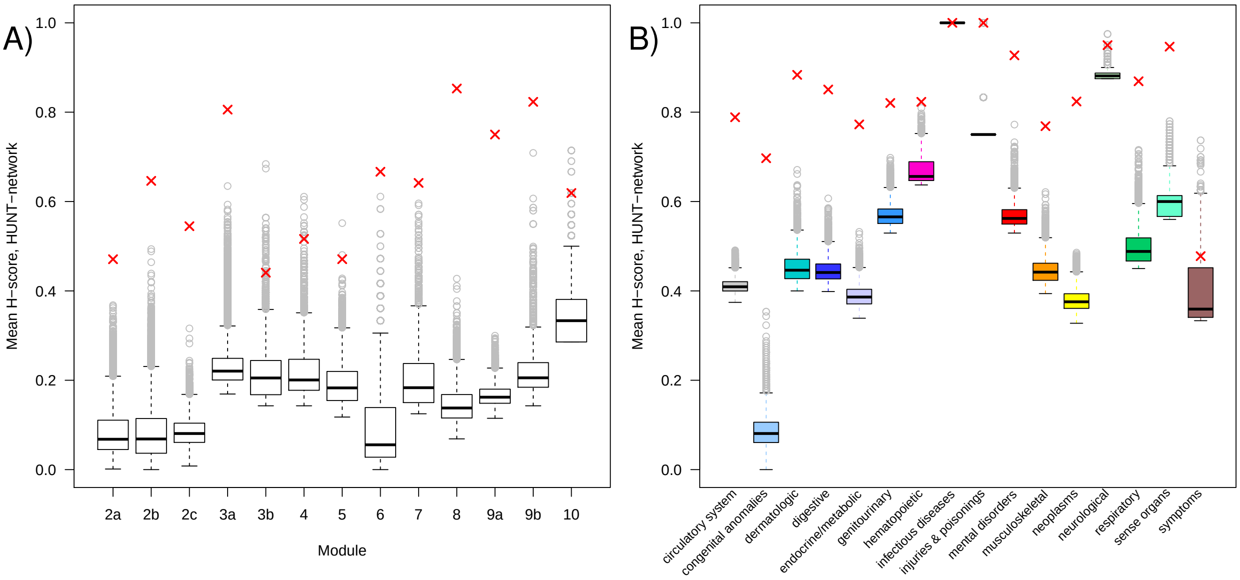

Fig. S4: Mean H-score of HUNT sub-PheNet compared to 10^4 random networks. Mean H score across the 13 largest modules A) and across the 16 phenotype categories B). The red x-es shows the results from the HUNT sub-PheNet, while the boxes with whiskers and outliers shows the results from 10^4 simulated networks.

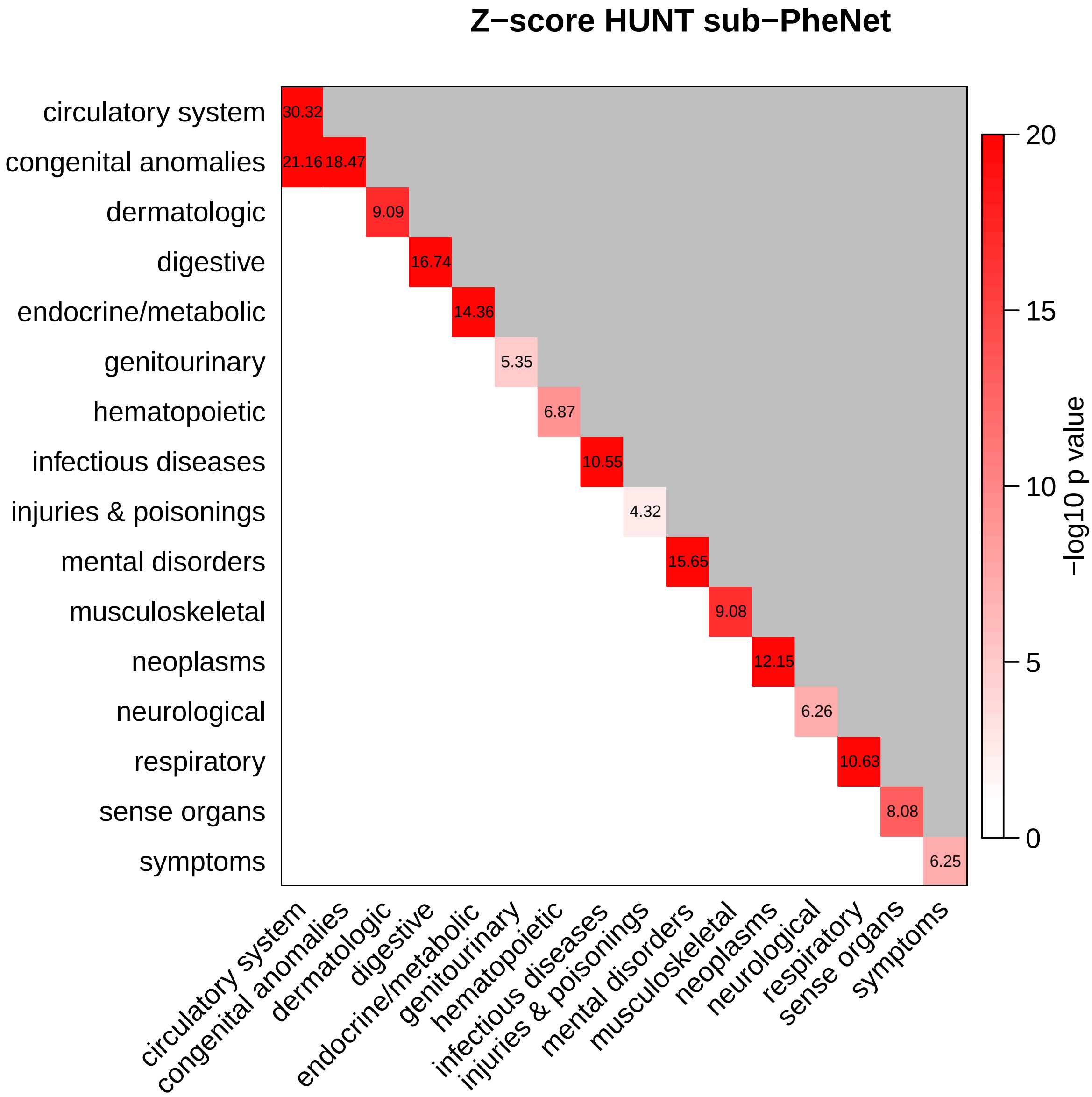

Fig. S5: Z-score of overlap between categories in the HUNT sub-PheNet. Entries show the Z-score where the corresponding two-sided p-values adjusted for multiple testing (136 tests) have $p < 0.05$. These entries are colored based on the base-10 exponent of the p-value from the Z-test. Only entries with Bonferroni-significant associations are highlighted and shown with numeric values.

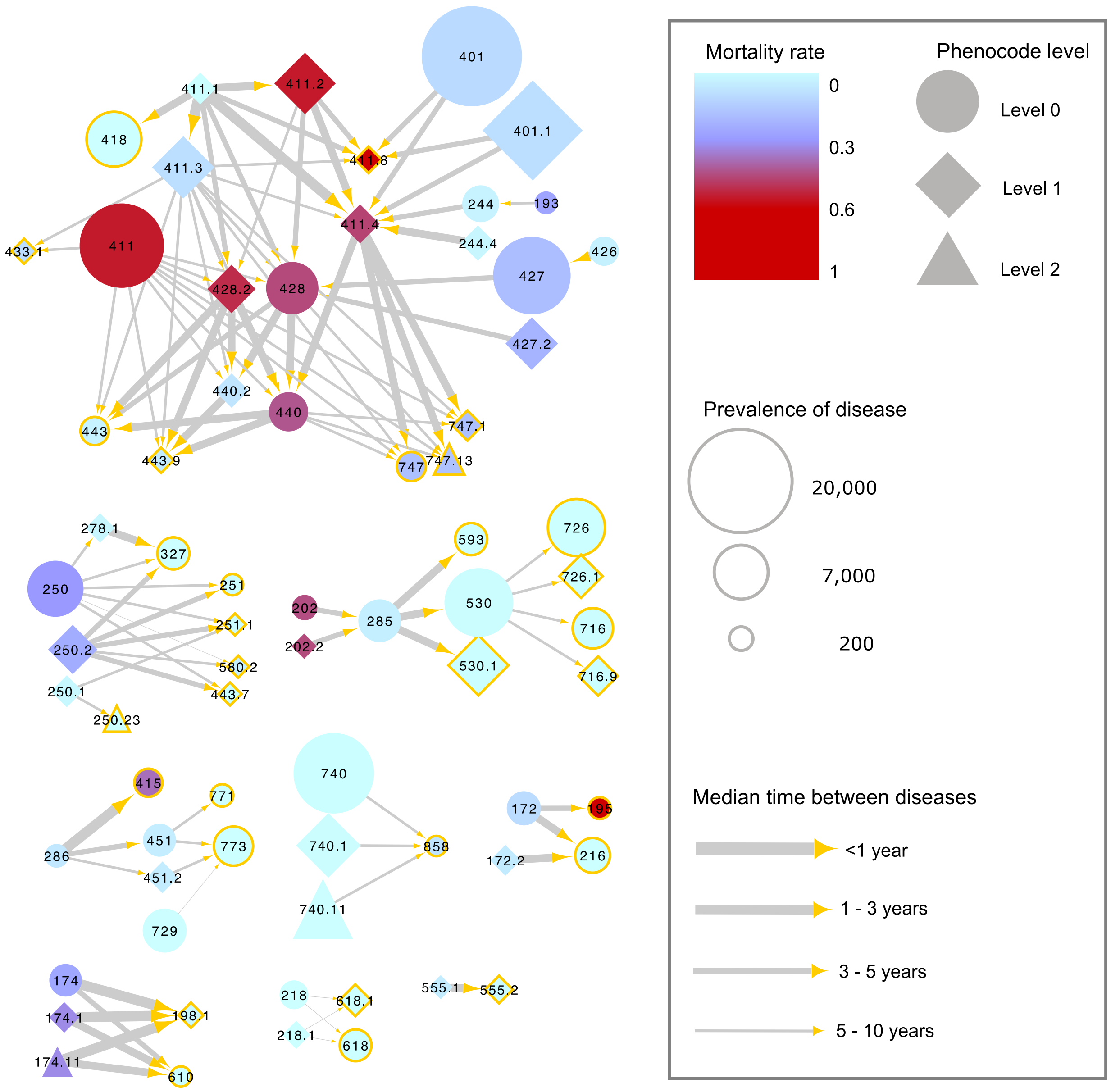

Fig. S6: The complete network of ordered pairs of diseases where the arrows show the directions of the disease histories, with thickness scaled by the median time between diagnoses. The size, shape, and color of nodes represents the prevalence, phenocode level, and mortality rate of the disease, respectively.
